# Re-emergence of Oropouche virus between 2023 and 2024 in Brazil: an observational epidemiological study

**DOI:** 10.1016/S1473-3099(24)00619-4

**Published:** 2025-02

**Authors:** Gabriel C Scachetti, Julia Forato, Ingra M Claro, Xinyi Hua, Bárbara B Salgado, Aline Vieira, Camila L Simeoni, Aguyda R C Barbosa, Italo L Rosa, Gabriela F de Souza, Luana C N Fernandes, Ana Carla H de Sena, Stephanne C Oliveira, Carolina M L Singh, Shirlene T S de Lima, Ronaldo de Jesus, Mariana A Costa, Rodrigo B Kato, Josilene F Rocha, Leandro C Santos, Janete T Rodrigues, Marielton P Cunha, Ester C Sabino, Nuno R Faria, Scott C Weaver, Camila M Romano, Pritesh Lalwani, José Luiz Proenca-Modena, William M de Souza

**Affiliations:** a Laboratory of Emerging Viruses, Department of Genetics, Evolution, Microbiology and Immunology, Institute of Biology, University of Campinas, Campinas, Brazil; b Department of Microbiology, Immunology, and Molecular Genetics, College of Medicine, University of Kentucky, Lexington, KY, USA; c Laboratory for Diagnosis and Control of Infectious Diseases in the Amazon, Instituto Leônidas e Maria Deane, Fiocruz Amazônia, Manaus, Brazil; d Laboratory of Infectious Diseases and Immunology, Instituto Leônidas e Maria Deane, Fiocruz Amazônia, Manaus, Brazil; e Universidade Federal do Amazonas, Manaus, Brazil; f Programa de Pós-Graduação Stricto Sensu em Imunologia Básica e Aplicada, Universidade Federal do Amazonas, Manaus, Brazil; g Laboratório Central de Saúde Pública do Ceará, Fortaleza, Brazil; h Centro Nacional de Inteligência Epidemiológica, Secretária de Vigilância em Saúde, Ministério da Saúde, Brasília, Brazil; i Instituto de Ciências Biológicas, Universidade Federal de Minas Gerais, Belo Horizonte, Minas Gerais, Brazil; j Laboratório Central de Saúde Pública do Acre, Rio Branco, Brazil; k Instituto de Medicina Tropical, Faculdade de Medicina da Universidade de São Paulo, São Paulo, Brazil; l Departamento de Patologia, Faculdade de Medicina da Universidade de São Paulo, São Paulo, Brazil; m MRC Centre for Global Infectious Disease Analysis, Department of Infectious Disease Epidemiology, School of Public Health, Imperial College London, London, UK; n Department of Zoology, University of Oxford, Oxford, UK; o Department of Microbiology and Immunology and World Reference Center for Emerging Viruses and Arboviruses, University of Texas Medical Branch, Galveston, TX, USA; p Hospital das Clínicas da Faculdade de Medicina da Universidade de São Paulo, São Paulo, Brazil

## Abstract

**Background:**

Oropouche virus is an arthropod-borne virus that has caused outbreaks of Oropouche fever in central and South America since the 1950s. This study investigates virological factors contributing to the re-emergence of Oropouche fever in Brazil between 2023 and 2024.

**Methods:**

In this observational epidemiological study, we combined multiple data sources for Oropouche virus infections in Brazil and conducted in-vitro and in-vivo characterisation. We collected serum samples obtained in Manaus City, Amazonas state, Brazil, from patients with acute febrile illnesses aged 18 years or older who tested negative for malaria and samples from people with previous Oropouche virus infection from Coari municipality, Amazonas state, Brazil. Basic clinical and demographic data were collected from the Brazilian Laboratory Environment Management System. We calculated the incidence of Oropouche fever cases with data from the Brazilian Ministry of Health and the 2022 Brazilian population census and conducted age–sex analyses. We used reverse transcription quantitative PCR to test for Oropouche virus RNA in samples and subsequently performed sequencing and phylogenetic analysis of viral isolates. We compared the phenotype of the 2023–24 epidemic isolate (AM0088) with the historical prototype strain BeAn19991 through assessment of titre, plaque number, and plaque size. We used a plaque reduction neutralisation test (PRNT_50_) to assess the susceptibility of the novel isolate and BeAn19991 isolate to antibody neutralisation, both in serum samples from people previously infected with Oropouche virus and in blood collected from mice that were inoculated with either of the strains.

**Findings:**

8639 (81·8%) of 10 557 laboratory-confirmed Oropouche fever cases from Jan 4, 2015, to Aug 10, 2024, occurred in 2024, which is 58·8 times the annual median of 147 cases (IQR 73–325). Oropouche virus infections were reported in all 27 federal units, with 8182 (77·5%) of 10 557 infections occurring in North Brazil. We detected Oropouche virus RNA in ten (11%) of 93 patients with acute febrile illness between Jan 1 and Feb 4, 2024, in Amazonas state. AM0088 had a significantly higher replication at 12 h and 24 h after infection in mammalian cells than the prototype strain. AM0088 had a more virulent phenotype than the prototype in mammalian cells, characterised by earlier plaque formation, between 27% and 65% increase in plaque number, and plaques between 2·4-times and 2·6-times larger. Furthermore, serum collected on May 2 and May 20, 2016, from individuals previously infected with Oropouche virus showed at least a 32-fold reduction in neutralising capacity (ie, median PRNT_50_ titre of 640 [IQR 320–640] for BeAn19991 *vs* <20 [ie, below the limit of detection] for AM0088) against the reassortant strain compared with the prototype.

**Interpretation:**

These findings provide a comprehensive assessment of Oropouche fever in Brazil and contribute to an improved understanding of the 2023–24 Oropouche virus re-emergence. Our exploratory in-vitro data suggest that the increased incidence might be related to a higher replication efficiency of a new Oropouche virus reassortant for which previous immunity shows lower neutralising capacity.

**Funding:**

São Paulo Research Foundation, Burroughs Wellcome Fund, Wellcome Trust, US National Institutes of Health, and Brazilian National Council for Scientific and Technological Development.

**Translation:**

For the Portuguese translation of the abstract see Supplementary Materials section.

## Introduction

Oropouche virus is an endemic and neglected arbovirus that causes Oropouche fever in central and South America.^1^ Oropouche fever is usually characterised by mild and self-limited, non-specific disease with headache, arthralgia, myalgia, nausea, vomiting, chills, and photophobia.^1^ However, some people can have more severe complications, such as haemorrhagic manifestations, meningitis, meningoencephalitis, pregnancy complications (eg, stillbirths and congenital disabilities, although further studies are needed to confirm frequency and mechanism), and death.^1^, ^2^, ^3^ Oropouche virus is probably transmitted in an enzootic cycle, mainly by *Culicoides paraensis* midges among pale-throated sloths (*Bradypus tridactylus*), non-human primates, and other wild mammals.^1^, ^4^ In urban areas, the human-amplified cycle appears to have been established on multiple occasions involving midges and humans, and potentially some mosquito species.^1^, ^4^ Neither licensed vaccines nor antiviral drugs are available to prevent Oropouche virus infection or to treat people with Oropouche fever.

**Research in context**

**Evidence before this study**

Oropouche virus, an arthropod-borne virus endemic in central and South America, causes Oropouche fever and is characterised by a debilitating and relapsing febrile illness, which in some cases can lead to neurological complications and death. Oropouche virus has caused outbreaks in the Amazon basin at least since the 1950s, but Oropouche fever is a neglected tropical disease with an unknown disease burden. In November, 2023, the number of Oropouche fever cases reported in Bolivia, Brazil, Colombia, and Peru increased. We searched PubMed, medRxiv, bioRxiv, and virological.org for studies published from Feb 5 up to Aug 30, 2024, using the terms “Oropouche virus”, “Oropouche fever”, “Oropouche reemergence”, “Oropouche emergence”, “Oropouche outbreak”, and “Oropouche epidemic”, with no language restrictions. Our search identified mostly reviews and comment articles and three studies describing the Oropouche fever cases between 2023 and 2024 in Brazil. These cases were caused by an Oropouche virus strain that is the result of a reassortment event, which occurs when two related orthobunyaviruses infect the same cell and exchange genetic segments. To our knowledge, there is little information about the Oropouche fever re-emergence between 2023 and 2024, and the factors driving this phenomenon are unknown.

**Added value of this study**

We investigated the spatial and temporal dynamics of Oropouche fever outbreaks in Brazil and showed that the 2023–24 re-emergence might be due to the new Oropouche virus reassortant, an isolate of which has better viral fitness than the prototype BeAn19991 strain because it replicates faster and to higher titres in mammalian cells within 24 h after infection. The novel isolate also had a plaque phenotype change that might suggest an increase in virulence. Additionally, this study shows that antibodies in the serum of individuals with a previous Oropouche virus infection were less efficient at neutralising the new 2023–24 Oropouche virus reassortment isolate than a historical Oropouche virus isolate.

**Implications of all the available evidence**

Our exploratory findings suggest that the new Oropouche virus reassortant circulating between 2023 and 2024 has the capacity to evade antibodies present in the serum of individuals previously infected with Oropouche virus, suggesting that this virus might escape population immunity and continue to circulate in the Amazon basin. Furthermore, the increase in viral fitness of the 2023–24 Oropouche virus reassortant might be contributing to the unprecedented epidemic spread of Oropouche fever cases during the 2023–24 epidemic in Latin America. Consequently, continued monitoring of Oropouche virus strains with an ability to escape immunity is crucial for epidemic preparedness. These results will be useful to inform public health interventions to anticipate and mitigate potential Oropouche virus epidemics.

Oropouche virus belongs to the species *Orthobunyavirus oropoucheense* in the *Orthobunyavirus* genus of the Peribunyaviridae family, and its genome consists of three single-stranded, negative-sense RNA molecules: small (S), medium (M), and large (L) segments.^5^ Like other multisegmented viruses, Oropouche virus can undergo reassortment events when two related orthobunyaviruses co-infect the same cell, resulting in progeny with mixed genomic segments from both parental strains.^6^ These events are important drivers of genetic divergence because they can alter vector competence and disease severity.^7^ For instance, three Oropouche virus reassortants have been identified in South America: Iquitos, Madre de Dios, and Perdões viruses.^8^, ^9^, ^10^

Oropouche virus infections have been documented in central and South America since the 1950s.^1^ Although the burden of Oropouche fever is unknown, some estimates suggest that over half a million human cases might have occurred since its first identification.^1^ Between November, 2023, and August, 2024, a substantial increase in Oropouche fever cases was observed in Brazil, Bolivia, Colombia, and Peru, and autochthonous cases were detected in Cuba.^11^ Here, we contextualise the Oropouche fever spread in Brazil from 2015 to 2024 and combine epidemiological, molecular, genomic, and serological analysis to describe and investigate the virological factors that might have contributed to Oropouche fever re-emergence.

## Methods

### Study design and participants

In this observational epidemiological study, we combined multiple data sources for Oropouche virus, including molecular, genomic, and serological data, with aggregated epidemiological data from Brazil. For molecular screening, we used residual serum samples from patients with acute febrile illnesses who tested negative for malaria, obtained from patients cared for in the public health system in Manaus City, Amazonas state, Brazil. Serum samples from individuals with previous Oropouche virus infection were obtained from residents of Coari municipality, Amazonas state, Brazil. All participants were aged 18 years or older, and written informed consent was obtained from all participants before sample collection. Health-care workers at the public health systems recruited and collected samples from individuals with acute febrile illness in Manaus City or individuals with previous Oropouche virus infection in Coari municipality, using convenience sampling. Sex and ethnicity data for the epidemiological dataset and the two populations that provided samples were obtained through self-report, with the option of male or female for sex and no predetermined options for ethnicity. All procedures followed the ethical standards of the responsible committee on human experimentation and were approved by the ethics committees from the Federal University of Amazonas (5.876.612 and 6.629.451) and Nilton Lins University (2.636.421). All animal procedures from this study were approved by the Ethics Committee on Animal Use of the University of Campinas (5171–1/2019, 6003–1/2022, and 5657–1/2020).

### Procedures

National epidemiological data of laboratory-confirmed cases were obtained from the Brazilian Ministry of Health. This dataset includes the aggregated number of Oropouche fever cases in Brazil from epidemiological week 1 (Jan 4–10) in 2015 to epidemiological week 32 in 2024 (Aug 4–10) and Oropouche fever cases per state. Incidences and sex–age comparison were calculated based on the 2022 Brazilian population census reported by the Brazilian Institute of Geography and Statistics.^12^

For the patients sampled in our molecular screening during 2024, basic clinical and demographic data were collected from the Brazilian Laboratory Environment Management System. Anonymised patient data were used in this study and are provided in the appendix 2 (p 13). Viral RNA was extracted from serum samples and tested for Oropouche virus RNA with a specific reverse transcription quantitative PCR (RT-qPCR) assay (appendix 2 p 1).^13^, ^14^ Details of the two RT-qPCR protocols used are shown in the appendix 2 (p 12).^13^, ^14^ Molecular analyses were conducted at Fiocruz, Manaus City, Brazil, and Laboratory of Emerging Viruses, University of Campinas, Campinas, Brazil. RT-qPCR-positive RNA samples were used for viral isolation in Vero CCL81 cells. Isolated viruses were confirmed and quantified by RT-qPCR and a focus-forming assay (appendix 2 pp 1–2). Viral isolates were sequenced with the nanopore platform (Oxford Nanopore Technologies, Oxford, UK) and subjected to phylogenetic and reassortment analyses with 474 publicly available Oropouche virus genomes from 1955 to 2024 (appendix 2 p 2) as well as evolutionary similarity analyses with a subset of 390 publicly available Oropouche virus genomes identified between 2022 and 2024, along with the two generated in this study. Additionally, we concatenated the three segments of genomes (476 strains, including two generated in this study) and screened for reassortment events using all available methods in Recombination Detection Program (version 5).

To characterise the phenotype of the 2023–24 epidemic isolate (AM0088) compared with the Oropouche virus strain BeAn19991 (ie, prototype), growth curve analysis and plaque assays were performed. Cells were infected at a multiplicity of infection of 0·1. Samples were harvested at 0, 3, 6, 12, and 24 h after infection, and titres were measured by focus-forming assay on Vero CCL81, Huh7, and U-251 cells. Viral replication dynamics and virulence in vitro were assessed by measuring plaque number and size at 36, 48, and 72 h after infection (appendix 2 p 3).^15^ Next, a plaque reduction neutralisation test (PRNT_50_) was performed to assess susceptibility to antibody neutralisation (appendix 2 p 3). AM0088 and BeAn19991 isolates were compared by use of serum samples from individuals with confirmed Oropouche virus infection from Coari municipality, Amazonas state, Brazil. Serum samples were tested by PRNT_50_ in Vero CCL81 cells after incubation with OROV strain BeAn19991 or AM0088.

Additionally, to evaluate cross-neutralisation between AM0088 and BeAn19991 isolates, 4-week-old C57BL/6 mice were inoculated intraperitoneally with either AM0088 (n=7) or BeAn19991 (n=5). After 4 weeks, blood was collected and a PRNT_50_ was performed to assess antigenic differences between the viral strains (appendix 2 p 3).

### Statistical analysis

The analyses were carried out in RStudio, version 4.4.0. R packages necessary for analysis and visualisation were readxl, tidyverse, dylyr, zoo, scales, lubridate, tmap, sf, ggplot2, ggbreak, ggpubr, and stringr. Sex and age groups of national Oropouche fever cases were compared by use of two-way ANOVA test, followed by Tukey's honest significant difference test if significant differences were identified. Phylogenetic trees for segments M (medium) and L (large) were constructed by use of the general time reversible plus frequency plus invariable sites plus gamma distribution with four categories (GTR +F + I + G4) nucleotide substitution model, while the TPM3 plus invariable sites plus gamma distribution with four categories (TPM3 + I + G4) model was used for segment S (small). Statistical analyses of the viral replication curves, the number of plaques, and plaque size were performed using the paired *t* test. To assess whether serum PRNT_50_ against the isolate of the 2023–24 epidemic were reduced compared with those against Oropouche virus strain BeAn19991 in individuals with a previous Oropouche virus infection, we used a binomial distribution model based on the proportion of samples in each of two categories (differences >0 and differences ≤0) as previously described.^16^ For analysis of differences in plasma neutralising antibody titres, mean PRNT_50_ for each sample was calculated as a mean of two technical duplicates, and the significance of the differences between group medians was determined by one-way ANOVA followed by Tukey's test of non-linear regression curves using GraphPad Prism software, version 8.2.1.

### Role of the funding source

The funders of the study had no role in the study design, data collection, data analysis, data interpretation, or writing of the report.

## Results

**Figure 1:**
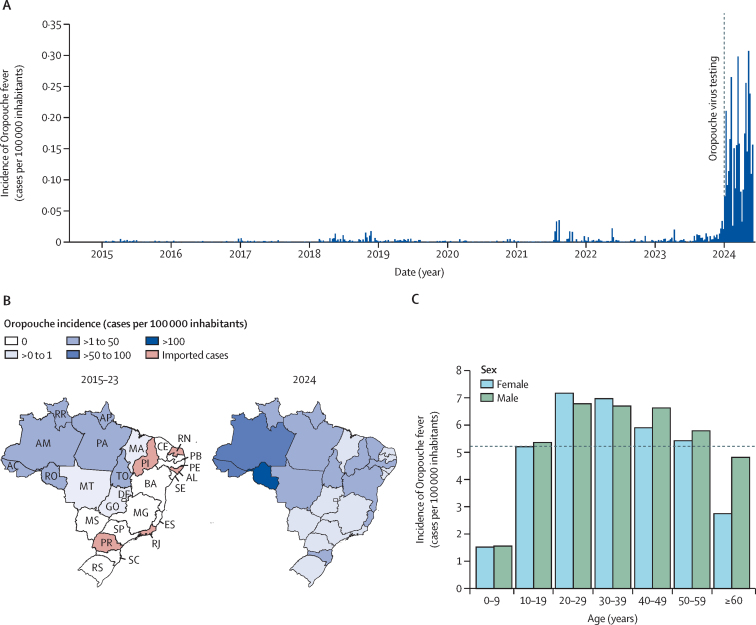
Spatial–temporal dynamics of Oropouche fever in Brazil, 2015–24 (A) Incidence of laboratory-confirmed Oropouche fever cases per epidemiological week in all 27 federal units (ie, 26 Brazilian states and the federal district), from epidemiological week 1 of 2015 (Jan 4–10) to epidemiological week 32 of 2024 (Aug 4–10). The dashed line indicates the implementation of Oropouche virus testing in 2024 across all central public health laboratories in Brazil by the Brazilian Ministry of Health.^17^ (B) Maps were coloured according to the incidence of laboratory-confirmed Oropouche fever cases by federal unit between January, 2015, and December, 2023, and between January and August, 2024. (C) Oropouche fever incidence based on the age–sex distribution of cases from 2015 to 2024. The dashed line indicates the national-level cumulative incidence. AC=Acre. AL=Alagoas. AM=Amazonas. AP=Amapá. BA=Bahia. CE=Ceará. DF=Distrito Federal (Federal District). ES=Espírito Santo. GO=Goiás. MA=Maranhão. MG=Minas Gerais. MS=Mato Grosso do Sul. MT=Mato Grosso. PA=Pará. PB=Paraíba. PE=Pernambuco. PI=Piauí. PR=Paraná. RJ=Rio de Janeiro. RN=Rio Grande do Norte. RO=Rondônia. RR=Roraima. RS=Rio Grande do Sul. SC=Santa Catarina. SE=Sergipe. SP=São Paulo. TO=Tocantins.

Between Jan 4, 2015, and Aug 10, 2024, 10 557 laboratory-confirmed Oropouche fever cases (5221 [49%] cases in women, 5327 [50%] cases in men, and nine [<1%] cases without sex data; 5795 cases were in people who self-reported as Pardo, 1853 in Asian people, 1526 in White people, 429 in Black people, 163 in Indigenous people, and 791 in people who did not declare race or ethnicity) were reported to the Brazilian Ministry of Health. Of these, 8639 (81·8%) were reported from January to August, 2024, representing 58·8 times more cases than the annual median of 147 cases (IQR 73–325) reported between 2015 and 2023 (figure 1A). Oropouche cases peaked annually between November and April, with 6124 (58·0%) of 10 557 cases reported during these months. North Brazil was the most affected region, contributing 8182 (77·5%) of 10 557 cases reported between 2015 and 2024 (figure 1B). Amazonas state reported the highest number of cases (3680 [34·9%]) and a cumulative incidence of 93·36 cases per 100 000 inhabitants from 2015 to 2024. Before 2024, Oropouche fever cases in Brazil primarily affected North Brazil. However, the 2024 epidemic resulted in widespread disease across Brazil, with autochthonous cases identified in all 27 federal units. These newly affected regions included Northeast, Southeast, Central-West, and South Brazil. The incidence of the disease in newly affected areas ranged from 0·03 to 11·00 cases per 100 000 inhabitants (figure 1B). Our analysis of the age–sex structure of Oropouche fever cases between 2015 and 2024 showed that female and male individuals aged 20–59 years had a higher incidence than the national-level cumulative incidence of 5·22 cases per 100 000 inhabitants (figure 1C). People aged 10–59 years had significantly higher incidence compared with younger people (ie, aged ≤9 years) and individuals aged 20–49 years had significantly higher incidence compared with older people (ie, ≥60 years; appendix 2 p 13). No significant sex differences were observed with two-way ANOVA test (p=0·27).

We next used RT-qPCR to investigate active Oropouche virus circulation in 93 patients (34 [37%] women and 59 [63%] men; 71 people self-reported as Pardo, nine as White, five as Black, four as Indigenous, three as Asian, and one did not declare race or ethnicity) with acute febrile illness who tested negative for malaria between Jan 1 and Feb 4, 2024. Oropouche virus RNA was detected in ten (11%) of 93 patients with acute febrile illness, with viral loads ranging from 1·65 × 10^3^ focus-forming units (FFU)/mL to 4·00 × 10^4^ FFU/mL. During molecular screening for Oropouche virus by use of two RT-qPCR protocols, we observed that samples from ten patients tested positive on one protocol targeting segment S^13^ but all tested negative with the other protocol targeting the same segment.^14^ We identified that mismatches between segment S of the 2023–24 Oropouche virus reassortant strains and the primers and probes likely affected Oropouche virus RNA detection (appendix 2 pp 4, 13). We also detected dengue virus RNA in two patients infected with serotypes 1 and 2. Chikungunya, Mayaro, and dengue viruses serotypes 3 and 4 were not detected. No cases of co-infection with Oropouche virus and other tested arboviruses were detected. We isolated Oropouche virus in Vero CCL-81 cells, with cytopathic effects observed within approximately 30 h after inoculation. Three blind passages were performed, and viral RNA was confirmed in seven (70%) of ten passaged strains by use of RT-qPCR on culture cell passages showing cytopathic effects. Next, we assessed viral titres using a focus-forming assay in all three passages and compared them with the original samples, showing the increased viral loads of passaged Oropouche virus isolates compared with the original patient samples (appendix 2 p 4).

**Figure 2:**
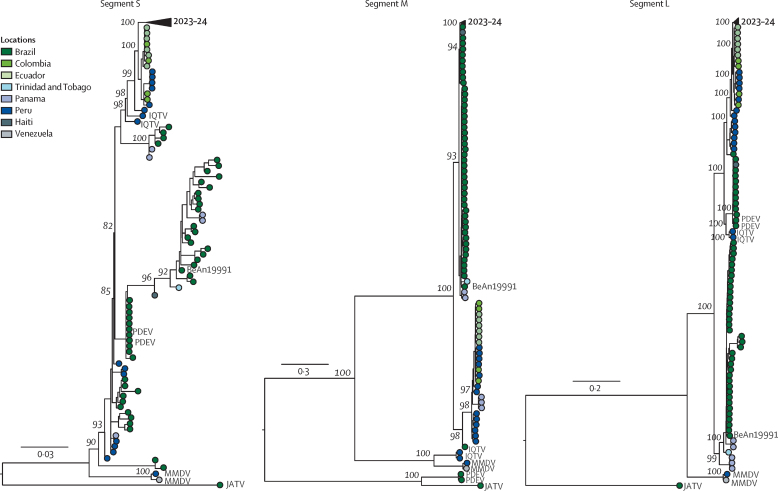
Phylogenetic analysis of Oropouche virus Maximum likelihood phylogenetic tree of 476 representative Oropouche virus genomes, including two new genomes generated in this study from serum samples of patients with febrile illnesses in Manaus City. Phylogenetic trees are shown for the S segment, M segment, and L segment. Tips are coloured according to the location country of each sample. Phylogenies were midpoint rooted for clarity of presentation. Scale bar indicates the evolutionary distance of substitutions per nucleotide site. Bootstrap values based on 1000 replicates are shown on principal nodes. The GenBank accession numbers of sequences used in this figure are shown in appendix 2 (pp 17–28). Detailed information on the collapsed clade with Oropouche virus reassortant strains circulating in 2023 and 2024 is provided in appendix 2 (pp 5–7). IQTV=Iquitos virus. JATV=Jatobal virus. MMDV=Madre de Dios virus. PDEV=Perdões virus.

We subsequently sequenced the nearly complete coding sequences of two Oropouche virus isolates (ie, AM0059 and AM0088). We obtained more than 90% coverage of two Oropouche virus genomes (all three segments) with a mean depth of coverage of more than or equal to 20-fold per nucleotide. We submitted sequences to GenBank (accession numbers PP992525–PP992530). Maximum likelihood phylogenetic analyses showed that the AM0059 and AM0088 strains clustered within a highly supported monophyletic clade (bootstrap support 100%) in all three genomic segments within 2023–24 strains in Amazonas, Acre, Roraima, and Rondônia states in Brazil and in Peru and Italy (figure 2; appendix 2 pp 5–7, 16–28). Based on the topology of phylogenetic trees estimated for the three Oropouche virus segments and the Recombination Detection Program (version 5)^18^ analysis with concatenated genomes (appendix 2 p 2), we identified that 2023–24 Oropouche virus strains resulted from a reassortment event, as previously described.^19^ This reassortant involved the M segment from a previously circulating Oropouche virus strain in North Brazil combined with S and L segments from a previous reassortant originating from Iquitos virus. Our identity analysis showed a median amino acid similarity of more than 99·9965% between Oropouche virus strains AM0059 and AM0088 and all three genomic segments of 390 reassortant Oropouche virus strains sampled from Brazil, Peru, and Italy during 2023 and 2024 (appendix 2 p 4). Furthermore, the L protein (ie, L segment) of AM0059 and AM0088 shared 95–96% amino acid identity with the Oropouche virus strain BeAn19991, representing 65 amino acid differences. The glycoprotein (ie, M segment) shared 98% identity with up to 31 amino acid differences (appendix 2 pp 13–16). No amino acid changes were found in the nucleoprotein (S segment) of AM0059 and AM0088 compared with BeAn19991 strain.

**Figure 3:**
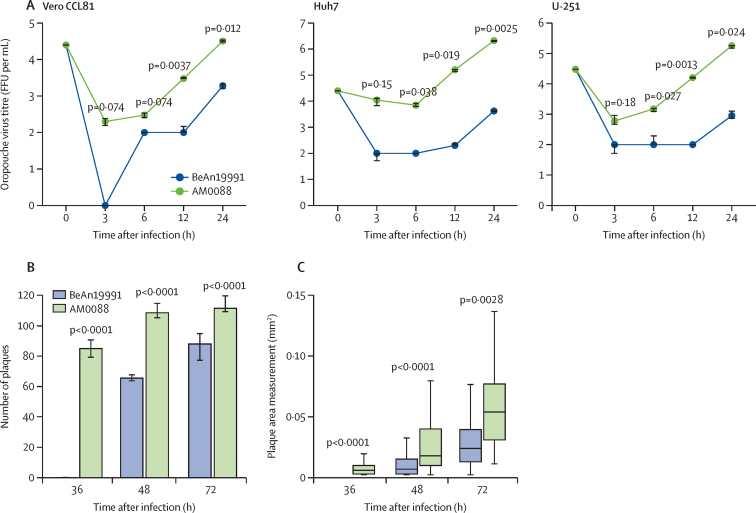
Characterisation in vitro of 2024 Oropouche virus reassortment strain (A) Viral replication properties of Oropouche virus strain AM0088 and Oropouche virus strain BeAn19991 in Vero CCL81, Huh7, and U-251 cell lines. The median is presented by the dot, and the upper and lower limits represent the 75th and 25th percentiles (ie, whiskers). (B) Number of plaques formed by Oropouche virus strains AM0088 and BeAn19991 after infection in Vero CCL81 cells (n=12 wells). The median is presented by the bar, and the upper and lower limits represent the 75th and 25th percentiles (ie, whiskers). (C) Size of plaques formed by Oropouche virus strains AM0088 and BeAn19991 after infection in Vero CCL81 cells (n≥118 plaques). The median is presented by the middle line, and the upper and lower limits represent the 75th and 25th percentiles (ie, whiskers). FFU=focus-forming units.

Next, we compared the replication kinetics of Oropouche virus strain AM0088, a genotypically representative strain of the 2023–24 Oropouche virus epidemic, and Oropouche virus strain BeAn19991 in Vero CCL81, Huh7, and U-251 cell lines. We identified a significantly higher replication of the AM0088 strain than of BeAn19991 in all cell lines at 12 h and 24 h after infection, with a median difference of 157 times (IQR 57–394) at these specified timepoints (figure 3A). We did not extend the viral replication curve beyond 24 h after infection due to the substantial cytopathic effect induced by Oropouche virus strain AM0088. The infected monolayer was substantially disrupted at 36 h after infection and completely destroyed at 48 h after infection. Furthermore, we observed that the AM0088 strain formed plaques at 36 h after infection, whereas BeAn19991 presented plaques at 48 h after infection. Additionally, the AM0088 strain formed 65% more plaques at 48 h after infection (109 plaques for AM0088 *vs* 66 plaques for BeAn19991) and 27% more plaques at 72 h after infection than BeAn19991 (112 plaques for AM0088 *vs* 88 plaques for BeAn19991; figure 3B). The AM0088 strain also had a distinctly larger plaque phenotype than BeAn19991, with 2·6-fold larger diameter at 48 h after infection (0·0180 mm^2^ *vs* 0·0070 mm^2^) and 2·4-fold larger diameter at 72 h after infection (0·0575 mm^2^ *vs* 0·0240 mm^2^; figure 3C).

**Figure 4:**
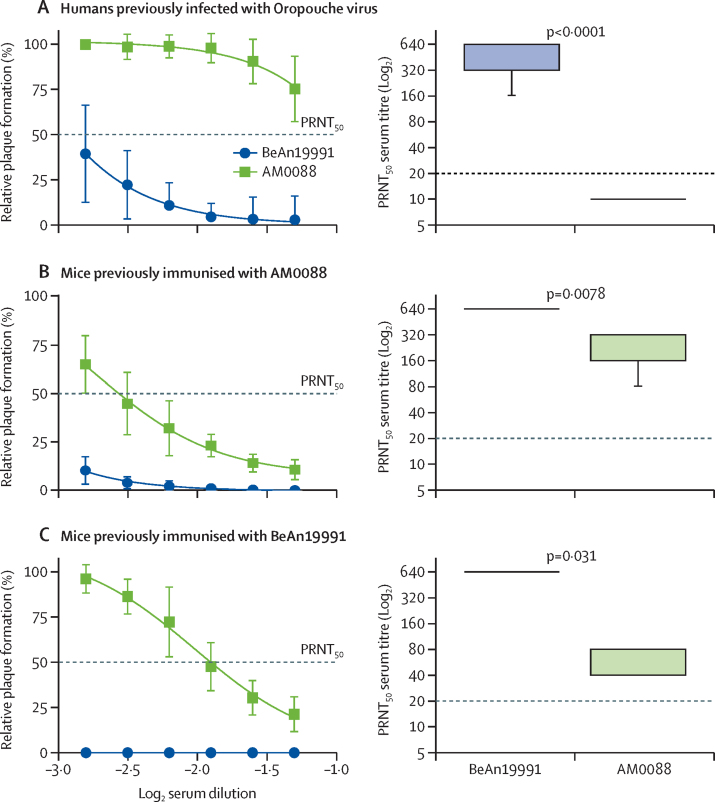
Neutralisation of Oropouche virus strains BeAn19991 and AM0088 by PRNT_50_ For relative plaque formation, each data point represents the mean of all serum samples for each group at each dilution level (shown as log_2_ serum dilution) and error bars represent SD. Dashed lines indicate the lower limit of detection (PRNT_50_=20) of the PRNT_50_ assay for samples with low or absent virus neutralisation capacity. The median titre is presented by the middle line, and the upper and lower limits represent the 75th and 25th percentiles (ie, whiskers). (A) Serum samples from individuals previously infected with Oropouche virus in Coari, Amazonas State, Brazil (n=22). (B) Serum samples were collected from C57BL/6 mice 28 days after infection with Oropouche virus strain AM0088 (n=7). (C) Serum samples were collected from C57BL/6 mice 28 days after infection with Oropouche virus strain BeAn19991 (n=5).

Subsequently, we investigated whether the AM0088 strain might escape from neutralisation by antibodies induced by previous Oropouche virus infection. For this analysis, we used serum samples collected on May 2 and May 20, 2016, from 22 individuals (16 [73%] women and six [27%] men; 12 self-reported as Pardo, one as Black and Indigenous, and nine did not declare race or ethnicity) previously infected with Oropouche virus in Coari municipality, Amazonas state (appendix 2 p 1). Using a PRNT_50_ assay, we assessed the ability of these serum samples to neutralise both the BeAn19991 and the AM0088 isolates. We identified that serum from individuals previously infected with Oropouche virus had a median PRNT_50_ titre for BeAn19991 of 640 (IQR 320–640), whereas the same serum had PRNT_50_ titres below the limit of detection (ie, <20) against AM0088 isolate (p<0·0001; figure 4A). These findings suggest a minimum of roughly 32-fold reduction in the neutralising capacity of antibodies from previously infected individuals to target the AM0088 compared with the BeAn19991 isolate.

Lastly, we conducted cross-neutralisation tests to explore the serological relationships between AM0088 and BeAn19991 isolates after experimental infection of C57BL/6 mice. The serum samples collected 28 days after mice were infected with the AM0088 strain showed a median homologous neutralising antibody titre of 320 (IQR 160–320) against the AM0088 strain and a titre of 640 (640–640) against BeAn19991 (p=0·0078; figure 4B). Additionally, the serum samples from mice that were immunised with BeAn19991 strain showed a median neutralising antibody titre of 640 (IQR 640–640) against the BeAn19991 strain and a titre of 80 (40–80) for the AM0088 strain (p=0·031; figure 4C). These results indicate that AM0088 reassortant is serologically distinct and significantly less neutralised by antibodies generated through infections with BeAn19991 and AM0088 itself.

## Discussion

This study provides a comprehensive assessment of Oropouche fever in Brazil from 2015 to 2024, focusing on the 2024 re-emergence, which had a 58·8-fold higher incidence than the annual median between 2015 and 2023. The substantial increase in Oropouche fever cases can be partly attributed to the increase of Oropouche virus surveillance in public health laboratories across Brazil, which was implemented nationwide in Brazil in January, 2024, after being limited to North Brazil region.^17^ We observed that Amazonas was the most affected Brazilian state by the 2023–24 Oropouche fever re-emergence, caused by a new Oropouche virus reassortant that subsequently spread to many Brazilian states.^3^, ^19^ Our phylogenetic analysis showed that new Oropouche virus reassortant strains sequenced in 2024 across Brazil, Peru, and Italy (ie, an infection in a returned traveller from Cuba*)*^2^ are nearly identical at the amino acid level. Our preliminary data suggest high neutralising antibodies from previous Oropouche virus infection might not efficiently neutralise the 2023–24 Oropouche virus reassortment. Similarly, a previous study using convalescent human serum samples and mouse antiserum samples showed that previous Oropouche virus infection does not protect against Iquitos virus, a reassortant identified in Peru in 1999.^20^ These preliminary findings emphasise the limited protective immunity against Oropouche virus and its reassortants, suggesting that individuals can be re-infected and develop clinical disease.

Our findings show that the 2023–24 Oropouche virus reassortant has significantly higher replicative competence in mammalian cells (ie, Vero CCL81, Huh7, and U-251) than the historical Oropouche virus strain BeAn19991 at 12 h and 24 h after infection. The extent to which this faster replication translates to enhanced Oropouche virus transmissibility by vectors is unclear, but it is plausible that this viral fitness might lead to an increase in viraemia in humans and reservoirs (eg, sloths), resulting in more efficient infection of vectors during blood feeding than with previous strains.^21^, ^22^ Our in-vitro data indicate that the 2023–24 Oropouche virus reassortant strain results from genotypic changes that might be associated with a new phenotype characterised by larger and numerous plaques, which suggests increased virulence compared with the prototype strain.^15^, ^23^ Therefore, this phenotypic change might partly explain the observed increase in Oropouche virus incidence between November, 2023, and July, 2024, but further investigation is necessary to elucidate the molecular mechanisms underlying the faster replication in vitro and altered plaque morphology with the new Oropouche virus reassortant compared with the prototype strain. Similarly, further studies with additional Oropouche virus isolates at lower passages than used in our study are necessary to confirm our findings about the 2023–24 Oropouche virus reassortant strain because multiple passages of the BeAn19991 strain isolated in Brazil in 1960 might have affected its virulence. Furthermore, investigation into antibody affinity, avidity, specificity, epitope recognition, and the influence of defective-to-infectious particle ratios will be important to understand the immune response to this reassortant.

Our analysis showed a higher incidence of Oropouche fever in 2024 within North Brazil, a historically endemic region with documented Oropouche virus circulation since the 1950s.^1^ This observation suggests that human populations in these areas continue to be exposed to Oropouche virus and might be susceptible to re-infection due to reduced neutralising antibody capacity against new epidemic Oropouche virus variants, such as the 2023–24 Oropouche virus reassortant. This hypothesis is based on the principle that previous Oropouche virus infection elicits a robust humoral response that can protect against homologous infection but not against novel reassortants, as previously described for Iquitos virus.^8^ This low level of protection is because the main target of the neutralising antibody response for orthobunyaviruses is the trimeric spikes in the glycoprotein encoded by the M segment,^24^ and consequently, change of this segment or mutations in the glycoprotein gene might lead to reduced neutralising capacity and binding of antibodies. Furthermore, the 2023–24 epidemiological and genomic surveillance data show a geographical expansion of Oropouche fever into previously non-endemic areas, including densely populated states in Brazil, such as Bahia, Piauí, Ceará, Espírito Santo, Minas Gerais, Rio de Janeiro, and Santa Catarina States.^3^ This spread emphasises the risk posed to the large, immunologically naive population across the Americas combined with the widespread availability of *C paraensis* and other potential Oropouche virus vectors from the southeastern USA to Uruguay.^25^ Consequently, the potential reduced neutralising antibody concentrations and putative increased viral fitness of the novel reassortant might contribute to re-infections in endemic areas, such as the Amazon basin, but also increase spread into new regions. For example, Cuba reported the first Oropouche fever cases between May and July, 2024, and a few cases were identified in Italy, Spain, Germany, and the USA among travellers returning from Cuba.^2^ This scenario could contribute to the introduction and establishment of Oropouche virus in the Americas and beyond, such as previously observed with dengue virus, Zika virus, and chikungunya virus.^26^

We observed that peaks of Oropouche virus cases in the epidemic waves occur mainly between November and March, which coincides with the rainy season in the Brazilian Amazon region. Wet and warm periods have been described as crucial drivers of the magnitude and seasonality of vector-borne virus transmission based on how they affect vector reproduction, survival, biting rates, and population density.^27^ Previous studies show that *C paraensis* has the greatest abundance in the rainy season, with temperatures between 30°C and 32°C and relative air humidity between 75% and 85%, with a substantial decrease in numbers during the dry season.^28^ However, the absence of information about the effects of climate and the weather on *C paraensis* and other potential Oropouche virus vectors prevents exploration of the potential effects of increased temperatures and the 2023–24 El Niño event as potential ecological factors associated with the Oropouche virus re-emergence. Additionally, our data indicate that Oropouche virus affected predominantly female and male adults aged 20–59 years. However, a comprehensive understanding of risk factors requires further exploration of ecological and individual-level determinants, including socioeconomic status, occupation, and deprivation.^29^

This study has several limitations. First, the absence of historical data from individuals with previous Oropouche virus infection prevents us from establishing the Oropouche virus strain involved and estimating the time since previous exposure. However, the higher neutralising antibody titres against BeAn19991 than against the novel reassortant suggest that previous infections in these individuals were likely caused by an Oropouche virus strain similar to BeAn19991. Second, neutralising antibodies against orthobunyaviruses primarily target the glycoproteins encoded by the M segment. The S and L segments of the 2023–24 Oropouche virus reassortant originated from Iquitos virus, but the M segment contains 31 amino acid substitutions when compared with BeAn1991. Therefore, it is plausible that these amino acid changes might have a more substantial effect on viral fitness in humans and vectors than the reassortment itself. However, further studies using reverse genetics techniques are necessary to elucidate the relative contributions of these factors. Third, our study focused on neutralising antibodies, but cellular immune responses mediated by T cells or functions mediated by Fc-mediated antibody effector response might also influence protection against disease.^30^, ^31^ Additional studies are required to clarify the protective role of previous Oropouche virus exposure against re-infection and find out the duration of protective immunity. Fourth, further research in patients with Oropouche fever and experimental studies using vector and animal models are required to confirm the viral fitness of this new Oropouche virus reassortant. Another limitation is that our study relied on variable health-care-seeking behaviours, and the previous absence of molecular diagnostic capabilities for Oropouche virus at the national level might have contributed to underestimating the burden of Oropouche fever in Brazil in previous years. Likewise, we were unable to identify the full extent of mild or asymptomatic cases due to the challenges of syndromic surveillance in regions that are co-endemic for dengue virus, Mayaro virus, and chikungunya virus, all of which cause similar clinical presentations to Oropouche fever.^26^, ^32^

In conclusion, our preliminary findings provide an important context about the dynamics and virological drivers of Oropouche virus and could inform future studies and public health policy focusing on strategies to mitigate the effects of new outbreaks and epidemics. For example, the 2023–24 Oropouche virus re-emergence emphasises the need for robust molecular surveillance strategies targeting at least two viral genome segments. Additionally, Oropouche virus infection should be considered in the differential diagnosis of febrile illnesses, neurological manifestations, deaths, and pregnancy complications in endemic countries and returning travellers from those countries. Further investigation is warranted to define the burden of severe Oropouche fever. These findings emphasise the need for public health preparedness strategies and the development of vaccines that provide broad protection against Oropouche virus and its reassortants to respond to future outbreaks effectively.

### Contributors

### Data sharing

The de-identified individualised data provided by the Brazilian Ministry of Health can be made available for research purposes. Any future research should be approved by a committee on human experimentation. The data can be provided on request to the corresponding authors.

## Declaration of interests

We declare no competing interests.

## Supplementary Materials

Portuguese translation of the abstract
