# Supplementary material for: Re-emergence of Oropouche virus between 2023 and 2024 in Brazil: an observational epidemiological study
Source: Lancet Infect Dis. 2025 Feb;25(2):166–75. doi: 10.1016/S1473-3099(24)00619-4 (PMC11779697; doi:10.1016/S1473-3099(24)00619-4)
Supplement: Portuguese translation of the abstract [file mmc1.pdf]

THE LANCET
Infectious Diseases

Supplementary appendix 1

This translation in Portuguese was submitted by the authors and we reproduce it as supplied. It has not been peer reviewed. *The Lancet*'s editorial processes have only been applied to the original in English, which should serve as reference for this manuscript.

Esta tradução em português foi submetida pelos autores e nós não fizemos quaisquer alterações. Esta versão não foi revista por pares. O processo editorial do The Lancet só foi aplicado à versão original em inglês, que deve servir como referência para este artigo.

Supplement to: Scachetti GC, Forato J, Claro IM, et al. Re-emergence of Oropouche virus between 2023 and 2024 in Brazil: an observational epidemiological study. *Lancet Infect Dis* 2024; published online Oct 15. https://doi.org/10.1016/S1473-3099(24)00619-4.

Reemergência do vírus Oropouche entre 2023 e 2024 no Brasil: um estudo epidemiológico observacional

Resumo

Contexto: Oropouche é um vírus transmitido por artrópodes, é tem causado surtos de febre Oropouche na América Central e do Sul desde a década de 1950. Este estudo investigou fatores virológicos que contribuem para o ressurgimento da febre Oropouche no Brasil entre 2023 e 2024.

Métodos: Neste estudo epidemiológico observacional, combinamos várias fontes de dados sobre a infecções pelo vírus Oropouche no Brasil e conduzimos caracterização *in vitro* e *in vivo*. Coletamos amostras de soro obtidas em Manaus, Amazonas, Brasil, de pacientes apresentando doença febril aguda com 18 anos ou mais que testaram negativo para malária, bem como amostras de pessoas com infecção prévia pelo vírus Oropouche do município de Coari, Amazonas, Brasil. Dados clínicos e demográficos básicos foram coletados do Sistema Gerenciador de Ambiente Laboratorial. Calculamos a incidência de casos de febre de Oropouche com dados do Ministério da Saúde do Brasil e do censo populacional brasileiro de 2022 e conduzimos análises de idade-sexo. Usamos PCR quantitativo de transcrição reversa para testar o RNA do vírus Oropouche em amostras, e posteriormente, realizamos sequenciamento e análise filogenética de isolados virais. Comparamos o fenótipo do isolado epidêmico de 2023–24 (AM0088) com a histórica cepa BeAn19991 (protótipo) por meio da avaliação do título, número de placas e tamanho da placa. Usamos teste de neutralização por redução de placas ($PRNT_{50}$) para avaliar a suscetibilidade do novo isolado e do isolado BeAn19991 à neutralização de anticorpos, tanto em amostras de soro de pessoas previamente infectadas com o vírus Oropouche quanto em sangue coletado de camundongos que foram inoculados com uma das cepas.

Resultados: 8639 (81,8%) de 10557 casos de febre de Oropouche foram confirmados por métodos laboratoriais de 1º de janeiro de 2015 a 10 de agosto de 2024, ocorreram em 2024, o que é 58,8 vezes a mediana anual de 147 casos (IQR 73–325). Infecções pelo vírus Oropouche foram relatadas em todas as 27 unidades federais, com 8182 (77,5%) de 10557 infecções ocorrendo no Norte do Brasil. Detectamos o RNA do vírus Oropouche em dez (11%) de 93 pacientes com doença febril aguda entre 1º de janeiro e 4 de fevereiro de 2024, no Amazonas. Identificamos que o novo vírus Oropouche *reassortant* associado à epidemia de 2023–24 teve uma replicação significativamente maior em 12 h e 24 h após a infecção em células de mamíferos do que a cepa protótipo. A cepa AM0088 exibiu um fenótipo mais virulento do que o protótipo em células de mamíferos, caracterizado por formação de placas mais precoce, entre 27% e 65% de aumento no número de placas e placas entre 2,4 vezes e 2,6 vezes maiores. Além disso, o soro coletado em 2 e 20 de maio de 2016, de indivíduos previamente infectados com o vírus Oropouche mostrou uma redução de pelo menos 32 vezes na capacidade de neutralização (ou seja, título $PRNT_{50}$ mediano de 640 [IQR 320–640] para BeAn 19991 *vs* <20 [ou seja, abaixo do limite de detecção] para AM0088) contra a cepa *reassortant* em comparação com o protótipo.

Interpretação: Essas descobertas fornecem uma avaliação compreensiva da febre Oropouche no Brasil e contribuem para um melhor entendimento do ressurgimento do vírus Oropouche em 2023–24. Nossos dados exploratórios *in vitro* sugerem que o aumento da incidência pode estar relacionado a uma maior eficiência de replicação de um novo vírus Oropouche *reassortant* para o qual a imunidade anterior mostra menor capacidade de neutralização.

Financiamento: Fundação de Amparo à Pesquisa do Estado de São Paulo, *Burroughs Wellcome Fund*, *Wellcome Trust*, *US National Institutes of Health*, e Conselho Nacional de Desenvolvimento Científico e Tecnológico.
