# Supplementary material for: Re-emergence of Oropouche virus between 2023 and 2024 in Brazil: an observational epidemiological study
Source: Lancet Infect Dis. 2025 Feb;25(2):166–75. doi: 10.1016/S1473-3099(24)00619-4 (PMC11779697; doi:10.1016/S1473-3099(24)00619-4)
Supplement: Supplementary appendix 2 [file mmc2.pdf]

THE LANCET
Infectious Diseases

Supplementary appendix 2

This appendix formed part of the original submission and has been peer reviewed. We post it as supplied by the authors.

Supplement to: Scachetti GC, Forato J, Claro IM, et al. Re-emergence of Oropouche virus between 2023 and 2024 in Brazil: an observational epidemiological study. *Lancet Infect Dis* 2024; published online Oct 15. https://doi.org/10.1016/S1473-3099(24)00619-4.

Supplementary appendix

Supplementary Information: Materials and Methods

Definition of cases

Oropouche fever laboratory-confirmed cases were defined as patients with one positive laboratory result for Oropouche virus (OROV), either by reverse-transcription quantitative polymerase chain reaction (RT-qPCR), immunoglobulin M (IgM) detection, immunoglobulin G (IgG), neutralization test, or hemagglutination inhibition. No clinical epidemiological cases were included in this study.

Serum samples of individuals with convalescence Oropouche virus infection

Blood samples from individuals with previous OROV infection were obtained by venipuncture in May 2016 from residents of Coari municipality, Amazonas State, Brazil. The previous OROV infection was confirmed by plaque reduction neutralization test value 50 ($PRNT_{50}$)[1]. All the samples used in this study were negative by RT-qPCR for OROV, Mayaro (MAYV), chikungunya (CHIKV), and dengue viruses (DENV), as described. All samples were stored at –80°C.

Real-time Quantitative Reverse Transcription-Polymerase Chain Reaction for Oropouche, *Chikungunya, Dengue, and Mayaro Viruses*

Viral RNA was extracted from the serum samples using the Maxwell HT Viral TNA Kit (Cat no. AX2340, Promega, USA) with the KingFisher Flex Purification System robot (Thermo Fisher Scientific, USA), following the manufacturer's instructions. The extracted RNA was then tested by real-time RT-qPCR targeting OROV[2,3], CHIKV[4], DENV serotypes 1 to 4[5,6], and MAYV[7] using the qPCR BIO Probe 1-Step Go Lo - ROX Kit (Cat no. PB25.11-03, PCR Biosystems, UK). Reactions were performed on QuantStudio 3 (Applied Biosystems, USA). The primers and probes used for viral detection are described (Appendix, p 12).

Oropouche virus isolation in cell culture

OROV isolation in culture cells was performed by inoculating Vero CCL-81 cells with ten serum samples that tested positive for OROV RNA using RT-qPCR (Appendix, p 13). Briefly, Vero CCL-81 cells were plated in 24-well plates at a concentration of $2{\cdot}5\times10^5$ cells per mL ($1{\cdot}25\text{x}10^5$ cells per well) in Minimum Essential Eagle's Medium (DMEM) supplemented with 10% fetal bovine serum (FBS), and 1% of penicillin of 10,000 units and 10,000 µg/mL streptomycin solution. Subsequently, the serum samples (n=10) were diluted 1:10 in DMEM, treated with 2% penicillin and streptomycin, and added to the monolayer. After a one-hour incubation at 37ºC for adsorption, DMEM supplemented with 5% FBS and treated with 1% penicillin and streptomycin was added to the monolayer for maintenance. The cells were kept at 37ºC with 5% CO_2 and monitored for 30 hours until the cytopathic effect (CPE) became visible on an optical microscope. Next, the supernatant was collected and subjected to an RT-qPCR assay[3] to confirm viral isolation, indicated by a decrease in the Ct-value. Viral stocks of the third-passage OROV strain AM0088 and the eighth-passage OROV strain BeAn 19991, both produced in Vero CCL-81 cells, were used for $PRNT_{50}$, virus replication curve, plaque phenotype assessment, and cross-neutralization antibody testing. The OROV strain BeAn 19991 used in these experiments was sequenced as previously described (Appendix p 2). No amino acid differences were found between our viral stock and the GenBank sequences (Accession nos. KP052850, KP052851, and KP052852)

Focus forming assay for Oropouche virus

A focus formation assay was performed for the titration of OROV-positive serum samples and isolates, as previously described elsewhere[8]. Briefly, the samples were serially diluted in an 8-fold series in DMEM treated with 1% penicillin and streptomycin solution. Next, 100 µL of the dilutions were transferred to 96-well plates containing Vero CCL-81 cells (5×10^4 cells per well) with 80% confluence, which were incubated for 1 hour at 37°C with 5% CO_2 for viral adsorption. Subsequently, 125 µL of DMEM containing 0·75% carboxymethylcellulose and 5% FBS was added to the wells, and the plates were incubated at 37°C with 5% CO_2 for 48 hours. Next, the cells were fixed with 70 µL of 4% paraformaldehyde solution (PFA) and incubated for 1 hour at 4°C. After removing the PFA, the cells were washed with phosphate-buffered saline (PBS). The cell monolayer was then blocked for 30 minutes with 150 µL of blotto. After blocking, the monolayers were washed with Perm/Wash Buffer (PBS supplemented with 0·1% BSA and 0·1% Triton X-100) and incubated with the polyclonal anti-OROV antibody (Cat no. VR-1228AF, ATTC, USA). After a second wash with PermWash, the monolayers were incubated with an anti-mouse IgG secondary antibody (Cat no. AP124P, Sigma-Aldrich, USA). Finally, after a final wash with Perm/Wash Buffer, the assay was revealed using the True-Blue Peroxidase substrate (Cat no. 5510-0030, KPL, USA) for 30 minutes.

Oropouche virus genome sequencing and analysis
OROV genome sequencing was performed with two viral isolates using the SMART-9N protocol with the MinION platform (Oxford Nanopore Technologies, UK), as previously described[9]. The generated raw FAST5 files were then basecalled, demultiplexed, and trimmed using Guppy version 9·4·1 (Oxford Nanopore Technologies, UK). The barcoded files were aligned to the OROV reference genome (GenBank accession no. KP691612, KP691622, and KP691623) using minimap2 v·2·17·r941[10] and converted into BAM files using SAMtools[11]. BCFtools[12] were employed for variant calling, followed by consensus sequence building. Genome regions with coverage below 20× were represented by the letter "N". BCFtools, Samtools stats, and Samtools depth[13] were applied to compute the genome statistics.

Phylogenetic analysis
The two novel OROV genomes with >90% coverage were generated and aligned with the non-redundant OROV strains with complete coding sequences available in the GenBank database as of August 14, 2024 (Appendix, pp 17-28). Then, we built a multiple sequence alignment (MSA) for each segment using MAFFT version 7·450[14], and manual adjustment was conducted using Geneious Prime 2023·0·4. A maximum likelihood (ML) phylogeny trees were performed using IQ-TREE version 2 under substitution nucleotide models determined by ModelFinder[15,16]. The ultrafast-bootstrap approach with 1,000 replicates was used to determine the statistical support for nodes for the ML phylogenies. The phylogenetic trees were visualized using Figtree 1·4·2 (http://tree.bio.ed.ac.uk/software/figtree/). The evolutionary divergence between all three segments of OROV strains AM0059 and AM0088 and 374 reassortant OROV strains sampled from Brazil, Peru, and Italy during 2023 and 2024 were calculated with p-distance using the maximum composite likelihood model[17]. Codon positions included were 1st, 2nd, 3rd, and noncoding. The results are represented by similarity in nucleotide and amino acid levels (Appendix, p 4). Additionally, we concatenated the three segments of genomes, and we screened for reassortment events using all available methods in RDP version 5[18] (Appendix, p 13).

Plaque reduction neutralization test for Oropouche virus
To compare the neutralizing antibody capacity of serum from individuals previously infected with OROV against OROV strains BeAn 19991 (prototype) and AM0088 strain (2023-2024 OROV reassortment), we performed a $PRNT_{50}$ as described elsewhere[1]. Briefly, we inactivated the

complement system by heating serum samples at 56ºC degrees, then we performed serial dilutions of each serum sample and incubated with a solution containing 2×10^3 PFU/mL for BeAn 19991 isolate, or 80 PFU/mL of the AM0088 isolate, both for 1 hour at 37°C. Subsequently, the virus-serum mixtures were added to pre-formed Vero CCL-81 cell monolayers and incubated for 1 hour at 37°C in a 5% CO_2 atmosphere. Next, we removed the inoculum and added 1 mL of DMEM containing 0·75% carboxymethylcellulose and 5% FBS was gently added to each well, and the plates were incubated at 37°C in a 5% CO_2 atmosphere for 3 days. Finally, the cells were fixed with 500 µL of 8% paraformaldehyde solution for 1 hour and stained with 1% methylene blue (Cat no. PHR3838, Sigma-Aldrich, USA) for 5 minutes. Plaque reduction was calculated as the average of values from two technical duplicates, corresponding to the percentages of the number of plaques counted compared to the positive control. These values were transformed to Log_2 for better visualization in the graph and subjected to a three-parameter nonlinear dose-response inhibition regression test.

Virus replication curves for Oropouche virus

To compare the viral fitness OROV strains BeAn 19991 (prototype) or AM0088 (2023-2024 OROV reassortment), we performed virus replication curves using Vero CCL-81 cells (African green monkey kidney), Huh7 cells (human liver carcinoma), and U-251 cells (Human glioblastoma astrocytoma). In summary, the cells were infected with OROV strains BeAn 19991 or AM0088 at a MOI of 0·1 for 1 hour at 37°C in a 5% CO_2 atmosphere. Then, we removed the inoculum, washed the cell monolayer three times using PBS, and added Minimum Essential Eagle's Medium (DMEM) supplemented with 10% fetal bovine serum (FBS), and 1% of penicillin of 10,000 units and 10,000 µg/mL streptomycin solution. At 3-, 6-, 12-, and 24-hours post-infection (hpi), we collected the cell culture supernatant and determined the infectious virus using FFA as described above[8]. All the experiments were conducted in triplicate.

Assessment of plaque phenotypes of Oropouche virus

To evaluate the plaque phenotypes generated by OROV strains BeAn 19991 or AM0088, we counted the number of plaques and measured the size of plaques at 36-, 48-, and 72-hours post-infection (hpi) for both OROV isolate in Vero CCL-81 cells. The number of plaques produced by each strain at each time point was determined by visual observation and counting. To measure the plaques, we illuminated the plates with a white bottom light and took photos using a Canon EOS Rebel T7i at a focal distance of 54 mm. All assay images were then imported into Fiji software version 2·15·1 for further analysis. We pre-processed and filtered the images to obtain a clearer resolution of the plaques. Next, we used the "Analyze Particles" feature to identify and measure the plaques, using the well diameter as the reference scale. Finally, any identified noise was manually removed from the images.

Cross-neutralization antibody test for Oropouche virus

To investigate antigenic differences between the OROV BeAn 19991 and AM0088 isolates, we conducted cross-neutralization assays. Briefly, two groups of four-week-old C57BL/6 mice were intraperitoneally (IP) inoculated with 1×10^6 PFU using a final volume of 100 µl. One group was inoculated with the BeAn 19991 isolate, and the other with AM0088 isolate. The mice were then kept under pathogen-free conditions at the biosafety level 2 animal facility of the Institute of Biology at the University of Campinas. Next, serum from these animals was collected 28 days post-infection. All animals did not show any signs of disease. Then, we performed the $PRNT_{50}$ to evaluate the capacity of neutralizing antibodies against the same OROV isolate (homologous) or different OROV isolate (heterologous).

Supplementary Figures

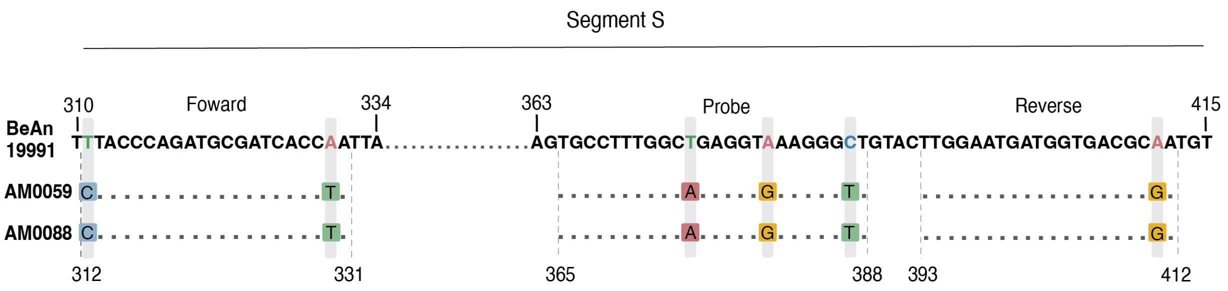

Supplementary Figure 1. Mismatches between the segment S of 2023-2024 OROV reassortment strains and the primers and probes previously described[2].

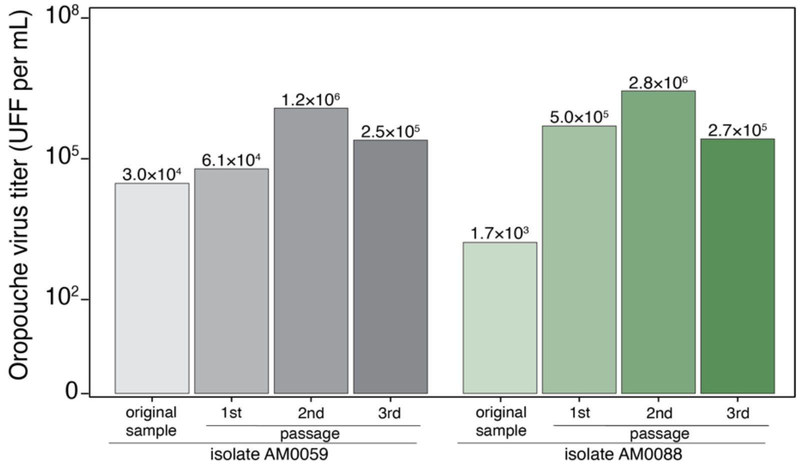

Supplementary Figure 2. Oropouche virus from the 2024 epidemic (AM0059 and AM0088) isolated from serum samples of Oropouche fever patients from Manaus City, Amazonas, Brazil. The isolation was also confirmed by focus forming assay, where it is possible to observe an increase in viral load.

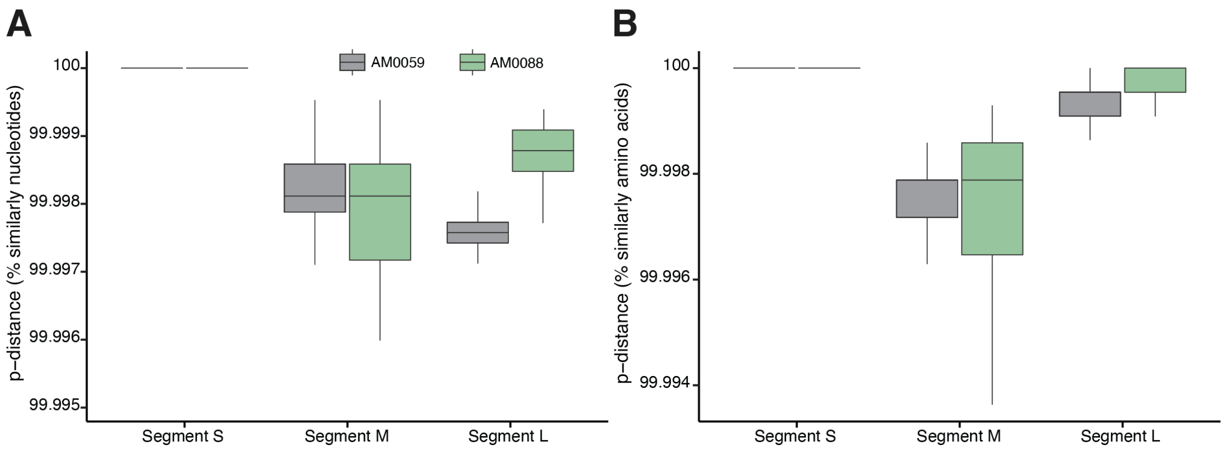

Supplementary Figure 3. Estimates of evolutionary similarity between OROV strains AM0059 and AM0088, and 374 reassortant OROV strains sampled from Brazil, Peru, and Italy during 2023 and 2024, using p-distance[17,19].

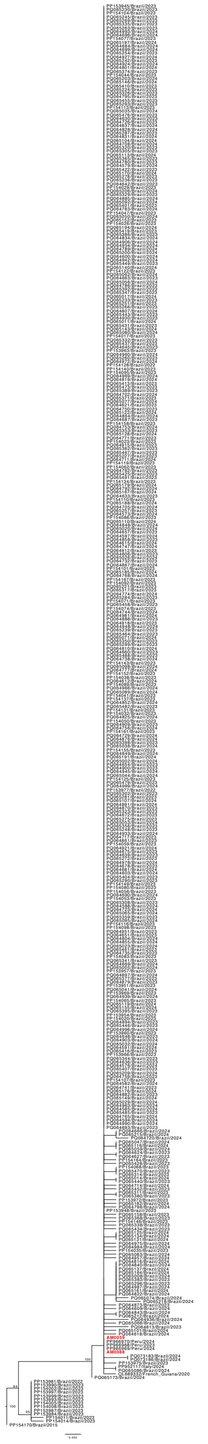

Supplementary Figure 4. Phylogenetic analysis of segment S of the Oropouche virus. This is a maximized clade of OROV reassortant genomes from 2023 to 2024 in Brazil, Peru, Italy, and French Guiana (n=392) presented as collapsed in Figure 2. The analysis includes OROV genomes from the AM0059 and AM0088 strains generated in this study (highlighted in red). Tip labels indicate GenBank accession number, country, and collection year. The tree is midpoint rooted for clarity, with bootstrap support values (1000 replicates) shown for major nodes**.** The scale bar indicates the evolutionary distance of substitutions per nucleotide site. Bootstrap values based on 1,000 replicates are shown on principal nodes. The GenBank accession numbers of sequences used in this figure are presented in (appendix p 17-28).

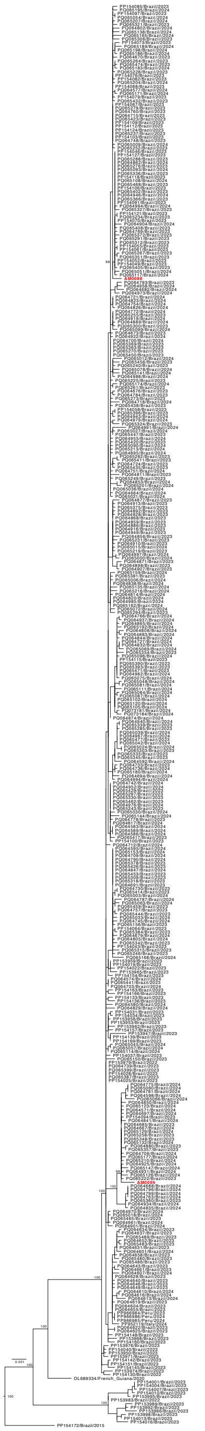

Supplementary Figure 5. Phylogenetic analysis of segment M of the Oropouche virus. This is a maximized clade of OROV reassortant genomes from 2023 to 2024 in Brazil, Peru, Italy, and French Guiana (n=392) presented as collapsed in Figure 2. The analysis includes OROV genomes from the AM0059 and AM0088 strains sequenced for this study (highlighted in red). Tip labels indicate GenBank accession number, country, and collection year. The tree is midpoint rooted for clarity, with bootstrap support values (1000 replicates) shown for major nodes**.** The scale bar indicates the evolutionary distance of substitutions

per nucleotide site. Bootstrap values based on 1,000 replicates are shown on principal nodes. The GenBank accession numbers of sequences used in this figure are presented in (appendix p 17-28).

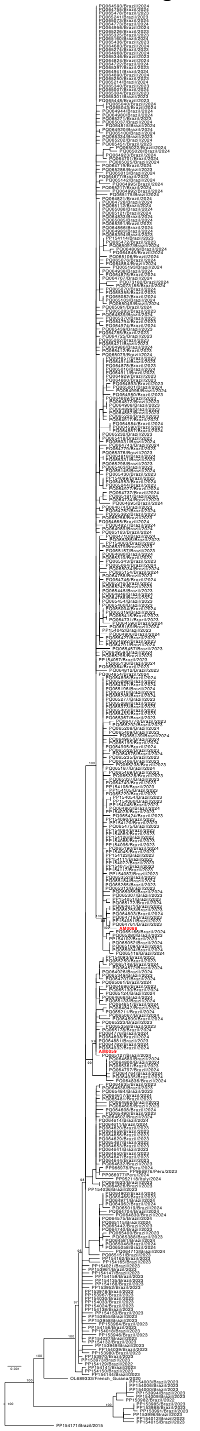

Supplementary Figure 6. Phylogenetic analysis of segment L of the Oropouche virus. This is a maximized clade of OROV reassortant genomes from 2023 to 2024 in Brazil, Peru, Italy, and French Guiana (n=392) presented as collapsed in Figure 2. The analysis includes OROV genomes from the AM0059 and AM0088 strains sequenced for this study (highlighted in red). Tip labels indicate GenBank accession number, country, and collection year. The tree is midpoint rooted for clarity, with bootstrap support values (1000 replicates) shown for major nodes**.** The scale bar indicates the evolutionary distance of substitutions

per nucleotide site. Bootstrap values based on 1,000 replicates are shown on principal nodes. The GenBank accession numbers of sequences used in this figure are presented in (appendix p 17-28).

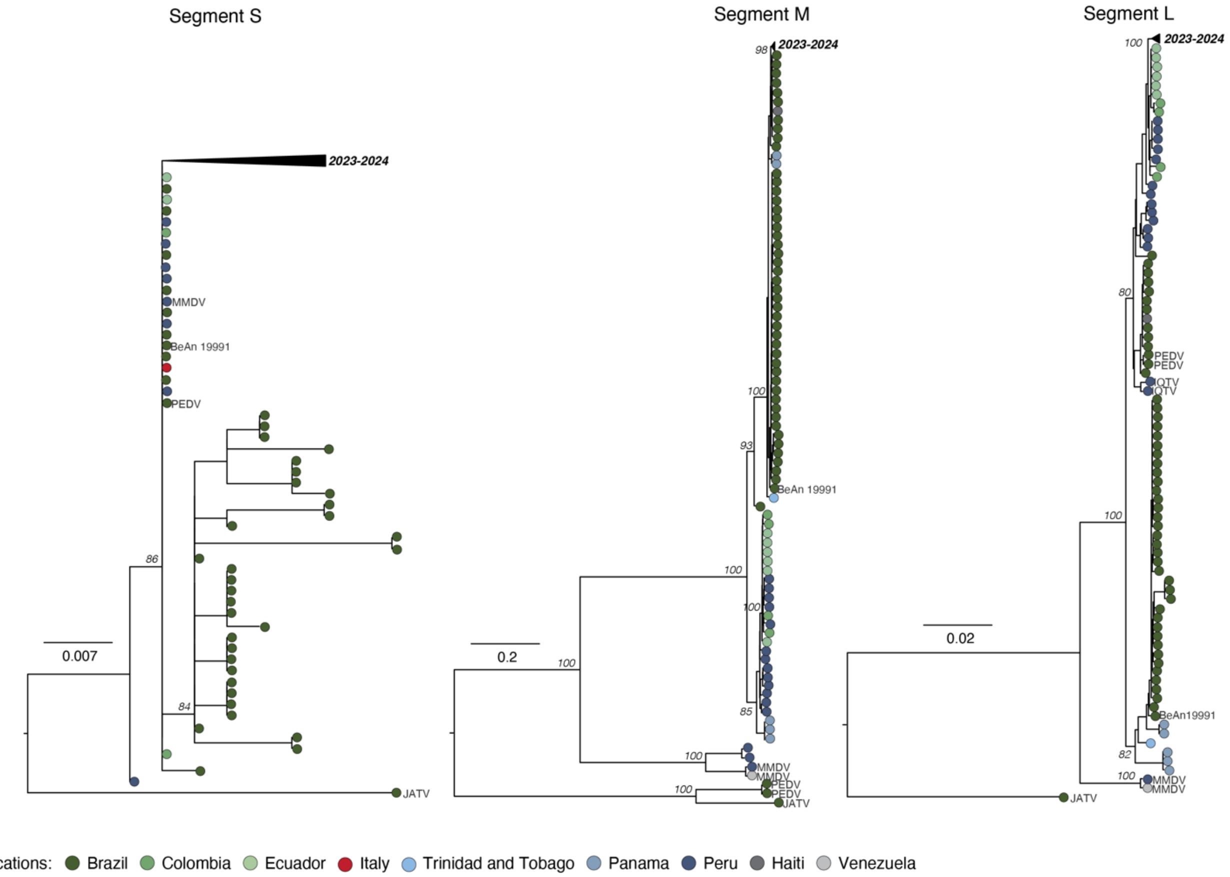

Supplementary Figure 7. Phylogenetic analysis of the Oropouche virus. Maximum likelihood phylogenetic tree of 476 representative OROV genomes at amino acid level, including two new genomes from Manaus City generated in this study. Phylogenetic trees are shown for the S segment (left), M segment (center), and L segment (right). Phylogenetic trees used the Q.bird (segment S), FLU+F+G4 (segment M) and FLU+F+I+G4 (segment L) amino acid substitution models.Tips are colored according to the location country of each sample. Phylogenies were midpoint rooted for clarity of presentation. Scale bar indicates the evolutionary distance of substitutions per amino acid site. Bootstrap values based on 1,000 replicates are shown on principal nodes. The GenBank accession numbers of sequences used in this figure are presented in appendix p 17-28. Detailed information on the collapsed clade with OROV reassortant strains circulating in 2023 and 2024 is provided in appendix 9-11.

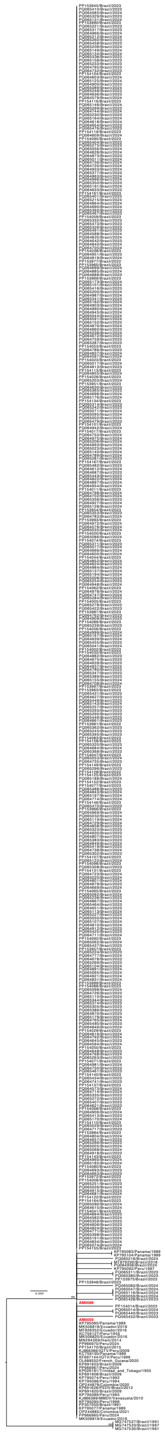

Supplementary Figure 8. Phylogenetic analysis of segment S (amino acid level) of the Oropouche virus. This is a maximized clade of OROV reassortant genomes from 2023 to 2024 in Brazil, Peru, Italy, and French Guiana (n=392) presented as collapsed in Supplementary Figure 6. The analysis includes OROV genomes from the AM0059 and AM0088 strains generated in this study (highlighted in red). Tip labels indicate GenBank accession number, country, and collection year. The tree is midpoint rooted for clarity, with bootstrap support values (1000 replicates) shown for major nodes**.** The scale bar indicates the evolutionary distance of substitutions per amino acid site. Bootstrap values based on 1,000 replicates are

shown on principal nodes. The GenBank accession numbers of sequences used in this figure are presented in (appendix p 17-28).

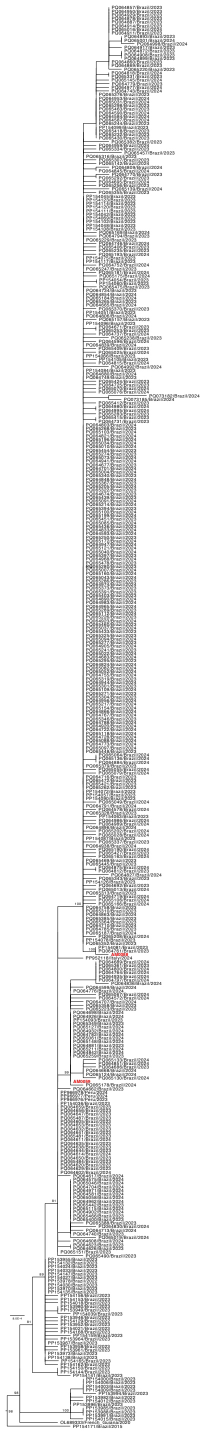

Supplementary Figure 9. Phylogenetic analysis of segment M (amino acid level) of the Oropouche virus. This is a maximized clade of OROV reassortant genomes from 2023 to 2024 in Brazil, Peru, Italy, and French Guiana (n=392) presented as collapsed in Supplementary Figure 6. The analysis includes OROV genomes from the AM0059 and AM0088 strains generated in this study (highlighted in red). Tip labels indicate GenBank accession number, country, and collection year. The tree is midpoint rooted for

clarity, with bootstrap support values (1000 replicates) shown for major nodes. The scale bar indicates the evolutionary distance of substitutions per amino acid site. Bootstrap values based on 1,000 replicates are shown on principal nodes. The GenBank accession numbers of sequences used in this figure are presented in (appendix p 17-28).

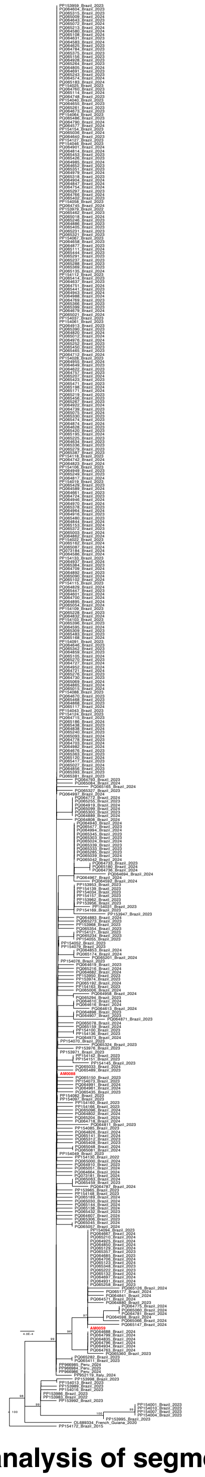

Supplementary Figure 10. Phylogenetic analysis of segment L (amino acid level) of the Oropouche virus. This is a maximized clade of OROV reassortant genomes from 2023 to 2024 in Brazil, Peru, Italy, and French Guiana (n=392) presented as collapsed in Supplementary Figure 6. The analysis includes OROV genomes from the AM0059 and AM0088 strains generated in this study (highlighted in red). Tip

labels indicate GenBank accession number, country, and collection year. The tree is midpoint rooted for clarity, with bootstrap support values (1000 replicates) shown for major nodes. The scale bar indicates the evolutionary distance of substitutions per amino acid site. Bootstrap values based on 1,000 replicates are shown on principal nodes. The GenBank accession numbers of sequences used in this figure are presented in (appendix p 17-28).

Supplementary Table

Supplementary Table 1. Primers and probes used for the detection of arboviruses in this study.

Virus	Sequences (5'→3')	Primers and probes	Target	Genome position	Ref.
OROV	TCCGGAGGCAGCATATGTG	Forward	S	98-116	3
	ACAACACCAGCATTGAGCACTT	Reverse	S	160-139	
	ATTTGAAGCTAGATACGG	Probe		118-136	
	TACCCAGATGCGATCACCAA	Forward	S	356-375	2
	TTGCGTCACCATCATTCCAA	Reverse	S	437-456	
	TGCCTTTGGCTGAGGTAAAGGGCT	Probe		409-433	
	TCGTCAACAAAACTCAACCACTTT	Forward	M	428-451	2
	GACCACAATTTACGGTTACATGCT	Reverse	M	525-548	
	TCGGGACAACTTCGACATCAGGCTG	Probe		459-483	
DENV1	GACACCACACCCTTTGGACAA	Forward	NS5	8586-8606	6
	CACCTGGGCTGTCACCTCCAT	Reverse	NS5	8692-8673	
	AGAGGGTGTTTAAAGAGAAAGTTGACACGCG	Probe		8608-8638	
DENV2	CAGGTTATGGCACTGTCACGAT	Forward	M	1605	5
	CCATCTGCAGCAACACCATCTC	Reverse	M	1583	
	CTCCGAGAACAGGCCTCGACTTCAA	Probe		1008	
DENV3	GGGAAAACCGTCTATCAATA	Forward	C	118-221	6
	CGCCATAACCAATTTCATTGG	Reverse	C	241-221	
	CACAGTTGGCGAAGAGATCTCAAGAGGA	Probe		174-202	
DENV4	TGAAGAGATTCTCAACCGGAC	Forward	C	187-207	6
	AATCCCTGCTGTTGGTGGC	Reverse	C	293-275	
	TCATCACGTTTTTGCGAGTCCTTTCCA	Probe		247-273	
CHIKV	AAAGGGCAAACTCAGCTTCAC	Forward	NSP1	874-894	4
	GCCTGGGCTCATCGTTATTC	Reverse	NSP1	961-942	
	CGCTGTGATACAGTGGTTTCGTGTG	Probe		899-923	
MAYV	AAGCTCTTCCTCTGCATTGC	Forward	NSP1	51-70	7
	TGCTGGAAACGCTCTCTGTA	Reverse1	NSP1	141-160	
	TGCTGGAAATGCTCTTTGTA	Reverse2		141-160	
	GCCGAGAGCCCGTTTTTAAAATCA	Probe		116-140	

Legend: OROV, Oropouche virus. DENV, dengue virus. CHIKV, chikungunya virus. MAYV, Mayaro virus. S, S segment. M, M segment. NS5, non-structural protein 5. M, matrix protein. C, capsid protein. NSP1, non-structural protein 1.

Supplementary Table 2. Anonymized patient information data for PCR-positive samples of Oropouche virus.

Group	ID	Sample collection	RT-PCR-SegS[3]	RT-PCR-SegM[3]	RT-PCR-SegS[2]	Viral isolate	Sequencing
Febrile illnesses	AM0004	12-Jan-2024	36·4	32·1	ND	yes	N/P
	AM0044	16-Jan-2024	37·0	ND	ND	no	N/P
	AM0045	19-Jan-2024	37·3	29·7	ND	yes	N/P
	AM0046	21-Jan-2024	35·2	28·0	ND	yes	N/P
	AM0059	21-Jan-2024	34·5	28·6	ND	yes	yes
	AM0063	20-Jan-2024	36·9	35·0	ND	no	N/P
	AM0064	21-Jan-2024	36·5	30·0	ND	yes	N/P
	AM0078	28-Jan-2024	34·2	26·0	ND	yes	N/P
	AM0088	30-Jan-2024	36·1	28·7	ND	yes	yes
	AM0093	22-Jan-2024	36·3	ND	ND	no	N/P

Seg S. segment S. SegM, segment M. N/P, not performed. ND, not detected.

Supplementary Table 3. Tukey's Honest Significant Difference test between age groups while accounting for the effect of sex.

	Age groups (years)						
**Age groups (years)**	**0 – 9**	**10 – 19**	**20 – 29**	**30 – 39**	**40 – 49**	**50 – 59**	**≥60**
**0 – 9**	-	$0{\cdot}00672^{**}$	$0{\cdot}00090^{***}$	$0{\cdot}00104^{**}$	$0{\cdot}00195^{**}$	$0{\cdot}00435^{**}$	$0{\cdot}07274^{ns}$
**10 – 19**	-	-	$0{\cdot}19955^{ns}$	$0{\cdot}25836^{ns}$	$0{\cdot}65290^{ns}$	$0{\cdot}99620^{ns}$	$0{\cdot}28336^{ns}$
**20 – 29**	-	-	-	$0{\cdot}99997^{ns}$	$0{\cdot}87065^{ns}$	$0{\cdot}36006^{ns}$	$0{\cdot}01488^{*}$
**30 – 39**	-	-	-	-	$0{\cdot}94532^{ns}$	$0{\cdot}45633^{ns}$	$0{\cdot}01849^{*}$
**40 – 49**	-	-	-	-	-	$0{\cdot}90146^{ns}$	$0{\cdot}04725^{*}$
**50 – 59**	-	-	-	-	-	-	$0{\cdot}15536^{ns}$
**≥60**	-	-	-	-	-	-	-

Statistical significance is $***p<0{\cdot}001$, $**p<0{\cdot}01$, and $*p<0{\cdot}05$; ns, not significant.

Supplementary Table 4. Reassortment analysis using RDP5[18] for Oropouche virus strains.

Detection methods	p-values*
RDP	3.94E-17
GENECONV	3.49E-17
Bootscan	1.48E-79
Maxchi	4.61E-259
Chimaera	3.18E-21
SiSscan	1.68E-44
3Seq	6.40E22

* OROV reassortant analysis of 476 representative genomes, including two new genomes from Manaus City generated in this study.

Supplementary Table 5. Amino acids change comparing Oropouche virus strain BeAn19991 to 2023-2024 OROV reassortment strains in glycoprotein.

Gene	Position	OROV strain BeAn19991	OROV reassortant strains 2020-2024 (n=392) *	Similarity (%)	Proteins
M	12	G	S	99	Gn
	66	K	R	99	Gn
	392	R	S	99	Non-structural protein
	393	I	T	100	Non-structural protein
	428	D	N	99	Non-structural protein
	446	V	I	100	Non-structural protein

514	S	N	99	Gc - Head domain
521	F	S	99	Gc - Head domain
587	F	L	100	Gc - Head domain
643	M	I	90	Gc - Head domain
732	L	V	99	Gc - Stalk subdomain 1
764	N	S	100	Gc - Stalk subdomain 1
822	A	T	99	Gc - Stalk subdomain 2
846	I	V	90	Gc - Stalk subdomain 2
1083	V	A	99	Gc - Core
1203	I	T	96	Gc - Core
1268	P	S	100	Gc - Core
1309	T	I	100	Gc - Core
1323	I	V	100	Gc - Core
1342	R	K	96	Gc - Core
1360	I	V	100	Gc - Core

* OROV reassortant genomes from Brazil, Peru, French Guiana, and Italy during 2015 and 2024, including OROV genomes from the AM0059 and AM0088 strains generated in this study.

Supplementary Table 6. Amino acids change comparing OROV strain BeAn19991 to 2023-2024 OROV reassortment strains (AM0059 and AM0088).

Gene	Position	BeAn19991 strain	AM0059 strain	AM0088 strain
M	12	G	S	S
	22	S	S	N
	66	K	R	R
	247	F	F	S
	276	F	I	I
	392	R	S	S
	393	I	T	T
	428	D	N	N
	446	V	I	I
	514	S	N	N
	521	F	S	S
	587	F	L	L
	643	M	I	I
	732	L	V	V
	764	N	S	S
	812	S	S	P
	822	A	T	T
	846	I	I	V
	957	I	I	V
	1083	V	A	A
	1203	I	T	T
	1268	P	S	S

	1309	T	I	I
	1323	I	V	V
	1342	R	K	K
	1360	I	V	V
L	135	T	A	A
	144	V	M	M
	201	T	A	A
	210	S	N	N
	215	A	S	S
	258	H	Q	Q
	263	T	A	A
	284	N	S	S
	303	M	I	I
	309	K	Q	Q
	313	N	S	S
	338	V	I	I
	339	N	S	S
	354	V	I	I
	372	I	V	V
	382	I	V	V
	415	L	F	F
	442	N	D	D
	458	I	T	T
	464	I	V	V
	558	M	I	I
	565	A	T	T
	580	A	T	T
	663	R	K	K
	677	S	A	A
	786	A	V	A
	788	R	Q	Q
	789	L	T	T
	790	S	V	V
	791	X	N	N
	794	V	I	I
	799	L	I	I
	800	Q	A	A
	801	E	R	R
	802	X	N	N
	850	R	K	K
	853	L	T	T
	854	R	K	K
	855	M	N	N
	856	I	D	D
	857	Q	A	A
	921	N	S	S

940	H	Y	Y
1035	N	S	S
1114	L	V	V
1159	I	T	T
1192	I	V	V
1314	S	N	N
1375	K	R	R
1436	D	N	N
1439	A	T	T
1505	V	I	I
1693	V	I	I
1758	V	I	I
1778	I	V	V
1911	R	Q	Q
1934	V	I	I
1942	V	I	I
1948	S	N	N
1961	I	V	V
1976	R	K	K
2056	G	E	E
2057	D	N	N
2171	K	R	R
2187	I	V	V

The positions refer to the competing coding sequences of OROV strain BeAn 19991 for the M segment (GenBank accession number KP052851) and the L segment (GenBank accession number KP052852).

Supplementary Table 7. Neutralizing capacity of serum previously infected with OROV in Coari municipality, Amazonas State, Brazil against the BeAn 19991 and the AM0088 isolates.

ID	BeAn 19991	AM0088
A 19	640	<20
A 20	640	<20
A 24	160	<20
A 33	640	<20
A 35	160	<20
A 77	320	<20
A 91	640	<20
A 111	320	<20
A 193	640	<20
A 206	640	<20
A 216	640	<20
A 304	320	<20
A 324	640	<20
A 336	640	<20
A 340	320	<20
A 351	640	<20
A 372	160	<20
A 375	320	<20
A 385	640	<20
A 389	320	<20
A 392	320	<20
A 369	640	<20

Supplementary Table 8. Genome sequences used in the phylogenetic analyses.

Isolate	Country	State	Host	Collection year	Accession GenBank numbers		
FPI21207	Peru	Loreto	Homo sapiens	2023	PP966980	PP966972	PP966964
FPI21246	Peru	Loreto	Homo sapiens	2024	PP966981	PP966973	PP966965
FPI21318	Peru	Loreto	Homo sapiens	2024	PP966982	PP966974	PP966966
FPI21339	Peru	Loreto	Homo sapiens	2024	PP966983	PP966975	PP966967
FPM01278	Peru	Madre de Dios	Homo sapiens	2023	PP966984	PP966976	PP966968
FPM01282	Peru	Madre de Dios	Homo sapiens	2024	PP966985	PP966977	PP966969
FPM01287	Peru	Madre de Dios	Homo sapiens	2024	PP966986	PP966978	PP966970
FPY01655	Peru	Loreto	Homo sapiens	2022	PP966987	PP966979	PP966971
IRCCS-SCDC_1/2024	Italy	Verona	Homo sapiens	2024	PP952119	PP952118	PP952117
-	Brazil	-	Homo sapiens	1991	PP357048	PP357049	PP357050
LVM_ILMD_ZDC388	Brazil	Rondonia	Homo sapiens	2023	PP153947	PP153946	PP153945
LVM_ILMD_ZDC125	Brazil	Rondonia	Homo sapiens	2023	PP153950	PP153949	PP153948
LVM_ILMD_ZDC089	Brazil	Rondonia	Homo sapiens	2023	PP153953	PP153952	PP153951
LVM_ILMD_ZCD208	Brazil	Rondonia	Homo sapiens	2023	PP153956	PP153955	PP153954
LVM_ILMD_ZCD166	Brazil	Rondonia	Homo sapiens	2023	PP153959	PP153958	PP153957
LVM_ILMD_ZCD155	Brazil	Rondonia	Homo sapiens	2023	PP153962	PP153961	PP153960
LVM_ILMD_ZCD142	Brazil	Rondonia	Homo sapiens	2023	PP153965	PP153964	PP153963
LVM_ILMD_ZCD123	Brazil	Rondonia	Homo sapiens	2023	PP153968	PP153967	PP153966
LVM_ILMD_ZCD121	Brazil	Rondonia	Homo sapiens	2023	PP153971	PP153970	PP153969
LVM_ILMD_ZCD117	Brazil	Rondonia	Homo sapiens	2023	PP153974	PP153973	PP153972
LVM_ILMD_ZCD103	Brazil	Rondonia	Homo sapiens	2023	PP153976	PP153980	PP153975
LVM_ILMD_ZCD067	Brazil	Rondonia	Homo sapiens	2022	PP153979	PP153978	PP153977
LACENRR_ILMD_0628MJG	Brazil	Roraima	Homo sapiens	2022	PP153983	PP153982	PP153981
LACENRR_ILMD_0156HM	Brazil	Roraima	Homo sapiens	2023	PP153986	PP153985	PP153984
LACENRR_ILMD_0153ARB	Brazil	Roraima	Homo sapiens	2023	PP153989	PP153988	PP153987
LACENRR_ILMD_0148ROS	Brazil	Roraima	Homo sapiens	2023	PP153992	PP153991	PP153990
LACENRR_ILMD_0058LAF	Brazil	Roraima	Homo sapiens	2023	PP153995	PP153994	PP153993
LACENRR_ILMD_0048LLS	Brazil	Roraima	Homo sapiens	2023	PP153998	PP153996	PP153997
LACENRR_ILMD_0048EPC	Brazil	Roraima	Homo sapiens	2023	PP154001	PP154000	PP153999
LACENRR_ILMD_0044DAS	Brazil	Roraima	Homo sapiens	2023	PP154004	PP154003	PP154002
LACENRR_ILMD_0033MSC	Brazil	Roraima	Homo sapiens	2023	PP154007	PP154006	PP154005
LACENRR_ILMD_0029MSM	Brazil	Roraima	Homo sapiens	2023	PP154010	PP154009	PP154008
LACENRR_ILMD_0026TSS	Brazil	Roraima	Homo sapiens	2023	PP154013	PP154012	PP154011
LACENRR_ILMD_0023SLR	Brazil	Roraima	Homo sapiens	2023	PP154016	PP154015	PP154014
LACENRO_ILMD_09	Brazil	Rondonia	Homo sapiens	2023	PP154019	PP154018	PP154017
LACENRO_ILMD_08	Brazil	Rondonia	Homo sapiens	2023	PP154022	PP154021	PP154020
LACENRO_ILMD_06	Brazil	Rondonia	Homo sapiens	2023	PP154025	PP154024	PP154023
LACENRO_ILMD_05	Brazil	Rondonia	Homo sapiens	2023	PP154028	PP154027	PP154026

LACENRO_ILMD_04	Brazil	Rondonia	Homo sapiens	2023	PP154031	PP154030	PP154029
LACENRO_ILMD_03	Brazil	Rondonia	Homo sapiens	2023	PP154034	PP154033	PP154032
LACENRO_ILMD_02	Brazil	Rondonia	Homo sapiens	2023	PP154037	PP154036	PP154035
LACENRO_ILMD_01	Brazil	Rondonia	Homo sapiens	2023	PP154040	PP154039	PP154038
LACENAM_ILMD_3896ERA	Brazil	Amazonas	Homo sapiens	2023	PP154043	PP154042	PP154041
LACENAM_ILMD_3022FSM	Brazil	Amazonas	Homo sapiens	2023	PP154046	PP154045	PP154044
LACENAM_ILMD_3020CAF	Brazil	Amazonas	Homo sapiens	2023	PP154049	PP154048	PP154047
LACENAM_ILMD_3012MC	Brazil	Amazonas	Homo sapiens	2023	PP154052	PP154051	PP154050
LACENAM_ILMD_3010MCF	Brazil	Amazonas	Homo sapiens	2023	PP154055	PP154054	PP154053
LACENAM_ILMD_3006MFP	Brazil	Amazonas	Homo sapiens	2023	PP154058	PP154057	PP154056
LACENAM_ILMD_3003JSS	Brazil	Amazonas	Homo sapiens	2023	PP154061	PP154060	PP154059
LACENAM_ILMD_3002JES	Brazil	Amazonas	Homo sapiens	2023	PP154064	PP154063	PP154062
LACENAM_ILMD_2999ICL	Brazil	Amazonas	Homo sapiens	2023	PP154067	PP154066	PP154065
LACENAM_ILMD_2997LA	Brazil	Amazonas	Homo sapiens	2023	PP154070	PP154069	PP154068
LACENAM_ILMD_2978ACS	Brazil	Amazonas	Homo sapiens	2023	PP154073	PP154072	PP154071
LACENAM_ILMD_2969MMS	Brazil	Amazonas	Homo sapiens	2023	PP154076	PP154075	PP154074
LACENAM_ILMD_2961GPP	Brazil	Amazonas	Homo sapiens	2023	PP154079	PP154078	PP154077
LACENAM_ILMD_2950JFS	Brazil	Amazonas	Homo sapiens	2023	PP154082	PP154081	PP154080
LACENAM_ILMD_2948RPS	Brazil	Amazonas	Homo sapiens	2023	PP154085	PP154084	PP154083
LACENAM_ILMD_2947MCM	Brazil	Amazonas	Homo sapiens	2023	PP154088	PP154087	PP154086
LACENAM_ILMD_2936GHS	Brazil	Amazonas	Homo sapiens	2023	PP154091	PP154090	PP154089
LACENAM_ILMD_2929LCF	Brazil	Amazonas	Homo sapiens	2023	PP154094	PP154093	PP154092
LACENAM_ILMD_2928KMG	Brazil	Amazonas	Homo sapiens	2023	PP154097	PP154096	PP154095
LACENAM_ILMD_2924GAA	Brazil	Amazonas	Homo sapiens	2023	PP154100	PP154099	PP154098
LACENAM_ILMD_2922CVM	Brazil	Amazonas	Homo sapiens	2023	PP154103	PP154102	PP154101
LACENAM_ILMD_2893AMM	Brazil	Amazonas	Homo sapiens	2023	PP154106	PP154105	PP154104
LACENAM_ILMD_2892TBT	Brazil	Amazonas	Homo sapiens	2023	PP154109	PP154108	PP154107
LACENAM_ILMD_2885RSM	Brazil	Amazonas	Homo sapiens	2023	PP154112	PP154111	PP154110
LACENAM_ILMD_2883KSA	Brazil	Amazonas	Homo sapiens	2023	PP154115	PP154114	PP154113
LACENAM_ILMD_2876RCS	Brazil	Amazonas	Homo sapiens	2023	PP154118	PP154117	PP154116
LACENAM_ILMD_2848DCS	Brazil	Amazonas	Homo sapiens	2023	PP154121	PP154120	PP154119
LACENAM_ILMD_2847DPS	Brazil	Amazonas	Homo sapiens	2023	PP154124	PP154123	PP154122
LACENAM_ILMD_2846GSM	Brazil	Amazonas	Homo sapiens	2023	PP154127	PP154126	PP154125
LACENAM_ILMD_2068TNM	Brazil	Amazonas	Homo sapiens	2022	PP154130	PP154129	PP154128
LACENAM_ILMD_0165GAS	Brazil	Amazonas	Homo sapiens	2023	PP154133	PP154132	PP154131
LACENAM_ILMD_0160ISS	Brazil	Amazonas	Homo sapiens	2023	PP154136	PP154135	PP154134
LACENAM_ILMD_0153JPS	Brazil	Amazonas	Homo sapiens	2023	PP154139	PP154138	PP154137
LACENAM_ILMD_0051JSL	Brazil	Amazonas	Homo sapiens	2023	PP154142	PP154141	PP154140
LACENAM_ILMD_0044NGF	Brazil	Amazonas	Homo sapiens	2023	PP154145	PP154144	PP154143
LACENAM_ILMD_0021CSO	Brazil	Amazonas	Homo sapiens	2023	PP154148	PP154147	PP154146
LACENAM_ILMD_0002AFS	Brazil	Amazonas	Homo sapiens	2023	PP154151	PP154150	PP154149

LACENAC_ILMD_0545	Brazil	Acre	Homo sapiens	2023	PP154154	PP154153	PP154152
LACENAC_ILMD_0504	Brazil	Acre	Homo sapiens	2023	PP154157	PP154156	PP154155
LACENAC_ILMD_0244	Brazil	Acre	Homo sapiens	2023	PP154160	PP154159	PP154158
LACENAC_ILMD_0151	Brazil	Acre	Homo sapiens	2023	PP154163	PP154162	PP154161
LACENAC_ILMD_0096	Brazil	Acre	Homo sapiens	2023	PP154166	PP154165	PP154164
LACENAC_ILMD_0093	Brazil	Acre	Homo sapiens	2023	PP154169	PP154168	PP154167
ILMD_TF29	Brazil	Amazonas	Homo sapiens	2015	PP154172	PP154171	PP154170
**0200178W**	Colombia	-	Homo sapiens	2020	OP244877	OP244878	OP244879
LET-352	Colombia	-	Homo sapiens	2021	OP244880	OP244881	OP244882
LET-882	Colombia	-	Homo sapiens	2021	OP244883	OP244884	OP244885
OROV/Saul/17225/2020	French Guiana	-	Homo sapiens	2020	OL689334	OL689333	OL689332
-	Brazil	-	Homo sapiens	2018	MT879228	MT879229	MT879230
-	Haiti	-	Homo sapiens	2014	MN264267	MN264268	MN264269
-	Ecuador	-	Homo sapiens	2016	MK506828	MK506823	MK506818
-	Ecuador	-	Homo sapiens	2016	MK506829	MK506824	MK506819
-	Ecuador	-	Homo sapiens	2016	MK506830	MK506825	MK506820
-	Ecuador	-	Homo sapiens	2016	MK506831	MK506826	MK506821
-	Ecuador	-	Homo sapiens	2016	MK506832	MK506827	MK506822
FCT00025/COL/2017	Colombia	-	Homo sapiens	2017	MK643117	MK643116	MK643115
BeH 543100	Brazil	-	Homo sapiens	1996	MG747505	MG747504	MG747503
BeH 389865	Brazil	-	Homo sapiens	1980	MG747508	MG747507	MG747506
BeH 390242	Brazil	-	Homo sapiens	1980	MG747511	MG747510	MG747509
BeH 472433	Brazil	-	Homo sapiens	1988	MG747514	MG747513	MG747512
BeH 472435	Brazil	-	Homo sapiens	1988	MG747517	MG747516	MG747515
BeH 421086	Brazil	-	Homo sapiens	1993	MG747520	MG747519	MG747518
BeAn 626990	Brazil	-	Callithrix sp.	2000	MG747523	MG747522	MG747521
BeAr 19886	Brazil	-	Ochlerotatus serratus	1960	MG747526	MG747525	MG747524
BeH 29086	Brazil	-	Homo sapiens	1961	MG747529	MG747528	MG747527
BeH 29090	Brazil	-	Homo sapiens	1961	MG747532	MG747531	MG747530
BeH 121923	Brazil	-	Homo sapiens	1967	MG747535	MG747534	MG747533
BeAr 136921	Brazil	-	Culex quinquefasciatus	1968	MG747538	MG747537	MG747536
BeAn 206119	Brazil	-	Bradypus tridactylus	1971	MG747541	MG747540	MG747539
BeAn 208402	Brazil	-	Bradypus tridactylus	1971	MG747544	MG747543	MG747542
BeAn 208819	Brazil	-	Bradypus tridactylus	1971	MG747547	MG747546	MG747545
BeH 355173	Brazil	-	Homo sapiens	1978	MG747550	MG747549	MG747548
BeAr 366927	Brazil	-	Culicoides paraensis	1979	MG747553	MG747552	MG747551
BeH 385591	Brazil	-	Homo sapiens	1980	MG747556	MG747555	MG747554
BeH 532314	Brazil	-	Homo sapiens	1994	MG747559	MG747558	MG747557
BeH 532422	Brazil	-	Homo sapiens	1994	MG747562	MG747561	MG747560
BeH 532490	Brazil	-	Homo sapiens	1994	MG747565	MG747564	MG747563

BeH 532500	Brazil	-	Homo sapiens	1994	MG747568	MG747567	MG747566
BeH 541140	Brazil	-	Homo sapiens	1994	MG747571	MG747570	MG747569
BeH 543629	Brazil	-	Homo sapiens	1996	MG747574	MG747573	MG747572
BeH 543760	Brazil	-	Homo sapiens	1996	MG747577	MG747576	MG747575
BeH 543857	Brazil	-	Homo sapiens	1996	MG747580	MG747579	MG747578
PPS 522 H 669314	Brazil	-	Homo sapiens	2003	MG747583	MG747582	MG747581
PPS 523 H 669315	Brazil	-	Homo sapiens	2003	MG747586	MG747585	MG747584
PMOH 682426	Brazil	-	Homo sapiens	2004	MG747589	MG747588	MG747587
PMOH 682431	Brazil	-	Homo sapiens	2004	MG747592	MG747591	MG747590
BeH 708139	Brazil	-	Homo sapiens	2006	MG747595	MG747594	MG747593
BeH 707287	Brazil	-	Homo sapiens	2006	MG747598	MG747597	MG747596
BeH 708717	Brazil	-	Homo sapiens	2006	MG747601	MG747600	MG747599
BeH 498913	Brazil	-	Homo sapiens	1990	MG747604	MG747603	MG747602
BeH 505768	Brazil	-	Homo sapiens	1991	MG747607	MG747606	MG747605
-	Ecuador	-	Homo sapiens	2016	MF926354	MF926353	MF926352
BeH759024	Brazil	-	Homo sapiens	2009	KP691603	KP691604	KP691605
BeH759021	Brazil	-	Homo sapiens	2009	KP691606	KP691607	KP691608
BeH759022	Brazil	-	Homo sapiens	2009	KP691609	KP691610	KP691611
BeH759025	Brazil	-	Homo sapiens	2009	KP691612	KP691613	KP691614
BeH759040	Brazil	-	Homo sapiens	2009	KP691615	KP691616	KP691617
BeH759529	Brazil	-	Homo sapiens	2009	KP691618	KP691619	KP691620
BeH759620	Brazil	-	Homo sapiens	2009	KP691621	KP691622	KP691623
BeH759146	Brazil	-	Homo sapiens	2009	KP691630	KP691631	KP691632
-	Trinidad and Tobago	-	-	1955	KP026179	KP026180	KP026181
-	Brazil	-	Bradypus tridactylus	1960	KP052850	KP052851	KP052852
-	Peru	-	Homo sapiens	1955	KC759125	KC759126	KC759127
-	Panama	-	Homo sapiens	1989	KC759128	KC759129	KC759130
AMA2291/H759582	Brazil	-	Homo sapiens	2009	OP407852	OP407853	OP407854
-	Peru	-	Homo sapiens	1992	KP795072	KP795073	KP795074
-	Panama	-	Homo sapiens	1989	KP795075	KP795076	KP795077
-	Panama	-	Homo sapiens	1989	KP795078	KP795079	KP795080
-	Panama	-	Homo sapiens	1989	KP795081	KP795082	KP795083
-	Peru	-	Homo sapiens	2008	KP795084	KP795085	KP795086
-	Peru	-	Homo sapiens	1995	KP795087	KP795088	KP795089
-	Peru	-	Homo sapiens	1997	KP795090	KP795091	KP795092
-	Peru	-	Homo sapiens	1998	KP795093	KP795094	KP795095
-	Peru	-	Homo sapiens	1994	KP795096	KP795097	KP795098
-	Peru	-	Homo sapiens	2000	KP795099	KP795100	KP795101
-	Panama	-	Homo sapiens	1999	KP795102	KP795103	KP795104
INHRR 17a-10	Venezuela	-	Cebus sp.	2010	KJ866391	KJ866390	KJ866389
FMD 1303	Peru	-	Homo sapiens	2007	KF697147	KF697145	KF697146

BeAn789726	Brazil	-	Callithrix penicillata	2012	KP691624	KP691625	KP691626
BeAn790177	Brazil	-	Callithrix penicillata	2012	KP691627	KP691628	KP691629
TVP-19261	Peru	-	Homo sapiens	2009	KJ866388	KJ866387	KJ866386
IQT9924	Peru	-	Homo sapiens	1999	KF697142	KF697143	KF697144
HAM_ILMD_24100006RST	Brazil	Amazonas	Homo sapiens	2024	PQ064571	PQ064572	PQ064573
HAM_ILMD_24100008MCS	Brazil	Amazonas	Homo sapiens	2024	PQ064574	PQ064575	PQ064576
HAM_ILMD_24100011GVC	Brazil	Amazonas	Homo sapiens	2024	PQ064577	PQ064578	PQ064579
ILMD_24100018VCN	Brazil	Amazonas	Homo sapiens	2024	PQ064580	PQ064581	PQ064582
ILMD_24100019	Brazil	Amazonas	Homo sapiens	2024	PQ064583	PQ064584	PQ064585
ILMD_24100020	Brazil	Amazonas	Homo sapiens	2024	PQ064586	PQ064587	PQ064588
ILMD_24100021	Brazil	Amazonas	Homo sapiens	2024	PQ064589	PQ064590	PQ064591
ILMD_24100023	Brazil	Amazonas	Homo sapiens	2024	PQ064592	PQ064593	PQ064594
ILMD_24100028	Brazil	Amazonas	Homo sapiens	2024	PQ064595	PQ064596	PQ064597
ILMD_24100033	Brazil	Amazonas	Homo sapiens	2024	PQ064598	PQ064599	PQ064600
LACENAC_ILMD_0024	Brazil	Amazonas	Homo sapiens	2024	PQ064601	PQ064602	PQ064603
LACENAC_ILMD_0044	Brazil	Acre	Homo sapiens	2023	PQ064604	PQ064605	PQ064606
LACENAC_ILMD_0047	Brazil	Amazonas	Homo sapiens	2024	PQ064607	PQ064608	PQ064609
LACENAC_ILMD_0178	Brazil	Acre	Homo sapiens	2024	PQ064610	PQ064611	PQ064612
LACENAC_ILMD_0182	Brazil	Acre	Homo sapiens	2024	PQ064613	PQ064614	PQ064615
LACENAC_ILMD_0185	Brazil	Acre	Homo sapiens	2024	PQ064616	PQ064617	PQ064618
LACENAC_ILMD_0543	Brazil	Acre	Homo sapiens	2023	PQ064619	PQ064620	PQ064621
LACENAC_ILMD_0633	Brazil	Acre	Homo sapiens	2023	PQ064622	PQ064623	PQ064624
LACENAC_ILMD_0650	Brazil	Acre	Homo sapiens	2023	PQ064625	PQ064626	PQ064627
LACENAC_ILMD_0700	Brazil	Acre	Homo sapiens	2023	PQ064628	PQ064629	PQ064630
LACENAC_ILMD_0733	Brazil	Acre	Homo sapiens	2023	PQ064631	PQ064632	PQ064633
LACENAC_ILMD_0779	Brazil	Acre	Homo sapiens	2023	PQ064634	PQ064635	PQ064636
LACENAC_ILMD_0977	Brazil	Acre	Homo sapiens	2023	PQ064637	PQ064638	PQ064639
LACENAC_ILMD_1279	Brazil	Acre	Homo sapiens	2023	PQ064640	PQ064641	PQ064642
LACENAC_ILMD_1729	Brazil	Acre	Homo sapiens	2023	PQ064643	PQ064644	PQ064645
LACENAC_ILMD_1730	Brazil	Acre	Homo sapiens	2023	PQ064646	PQ064647	PQ064648
LACENAC_ILMD_1751	Brazil	Acre	Homo sapiens	2023	PQ064649	PQ064650	PQ064651
LACENAC_ILMD_5561	Brazil	Acre	Homo sapiens	2023	PQ064652	PQ064653	PQ064654
LACENAC_ILMD_7053	Brazil	Acre	Homo sapiens	2023	PQ064655	PQ064656	PQ064657
LACENAC_ILMD_7055	Brazil	Acre	Homo sapiens	2023	PQ064658	PQ064659	PQ064660
LACENAC_ILMD_7062	Brazil	Acre	Homo sapiens	2023	PQ064661	PQ064662	PQ064663
LACENAC_ILMD_DSS	Brazil	Amazonas	Homo sapiens	2024	PQ064664	PQ064665	PQ064666
LACENAM_ILMD_0001	Brazil	Amazonas	Homo sapiens	2024	PQ064667	PQ064668	PQ064669
LACENAM_ILMD_0002MAC	Brazil	Amazonas	Homo sapiens	2023	PQ064670	PQ064671	PQ064672
LACENAM_ILMD_0002WSC	Brazil	Amazonas	Homo sapiens	2023	PQ064673	PQ064674	PQ064675
LACENAM_ILMD_0003GFA	Brazil	Amazonas	Homo sapiens	2023	PQ064676	PQ064677	PQ064678
LACENAM_ILMD_0003GMB	Brazil	Amazonas	Homo sapiens	2024	PQ064679	PQ064680	PQ064681

LACENAM_ILMD_0004FVC	Brazil	Amazonas	Homo sapiens	2024	PQ064682	PQ064683	PQ064684
LACENAM_ILMD_0005FSF	Brazil	Amazonas	Homo sapiens	2023	PQ064685	PQ064686	PQ064687
LACENAM_ILMD_0006	Brazil	Amazonas	Homo sapiens	2024	PQ064688	PQ064689	PQ064690
LACENAM_ILMD_0006MAP	Brazil	Amazonas	Homo sapiens	2023	PQ064691	PQ064692	PQ064693
LACENAM_ILMD_0007LSM	Brazil	Amazonas	Homo sapiens	2024	PQ064694	PQ064695	PQ064696
LACENAM_ILMD_0007MFS	Brazil	Amazonas	Homo sapiens	2024	PQ064697	PQ064698	PQ064699
LACENAM_ILMD_0008CSF	Brazil	Amazonas	Homo sapiens	2024	PQ064700	PQ064701	PQ064702
LACENAM_ILMD_0008GSM	Brazil	Amazonas	Homo sapiens	2024	PQ064703	PQ064704	PQ064705
LACENAM_ILMD_0008ORR	Brazil	Amazonas	Homo sapiens	2024	PQ064706	PQ064707	PQ064708
LACENAM_ILMD_0008VLR	Brazil	Amazonas	Homo sapiens	2024	PQ064709	PQ064710	PQ064711
LACENAM_ILMD_0009EPL	Brazil	Amazonas	Homo sapiens	2024	PQ064712	PQ064713	PQ064714
LACENAM_ILMD_0009RSS	Brazil	Amazonas	Homo sapiens	2023	PQ064715	PQ064716	PQ064717
LACENAM_ILMD_0009VSC	Brazil	Amazonas	Homo sapiens	2024	PQ064718	PQ064719	PQ064720
LACENAM_ILMD_0010	Brazil	Amazonas	Homo sapiens	2024	PQ064721	PQ064722	PQ064723
LACENAM_ILMD_0010FDC	Brazil	Amazonas	Homo sapiens	2023	PQ064724	PQ064725	PQ064726
LACENAM_ILMD_0011	Brazil	Amazonas	Homo sapiens	2024	PQ064727	PQ064728	PQ064729
LACENAM_ILMD_0011RMT	Brazil	Amazonas	Homo sapiens	2023	PQ064730	PQ064731	PQ064732
LACENAM_ILMD_0012APD	Brazil	Amazonas	Homo sapiens	2023	PQ064733	PQ064734	PQ064735
LACENAM_ILMD_0013	Brazil	Amazonas	Homo sapiens	2024	PQ064736	PQ064737	PQ064738
LACENAM_ILMD_0013EMP	Brazil	Amazonas	Homo sapiens	2023	PQ064739	PQ064740	PQ064741
LACENAM_ILMD_0015	Brazil	Amazonas	Homo sapiens	2024	PQ064742	PQ064743	PQ064744
LACENAM_ILMD_0015ELP	Brazil	Amazonas	Homo sapiens	2024	PQ064745	PQ064746	PQ064747
LACENAM_ILMD_0015ELV	Brazil	Amazonas	Homo sapiens	2023	PQ064748	PQ064749	PQ064750
LACENAM_ILMD_0016	Brazil	Amazonas	Homo sapiens	2024	PQ064751	PQ064752	PQ064753
LACENAM_ILMD_0020	Brazil	Amazonas	Homo sapiens	2024	PQ064754	PQ064755	PQ064756
LACENAM_ILMD_0022JSP	Brazil	Amazonas	Homo sapiens	2023	PQ064757	PQ064758	PQ064759
LACENAM_ILMD_0023VBS	Brazil	Amazonas	Homo sapiens	2023	PQ064760	PQ064761	PQ064762
LACENAM_ILMD_0026AHJ	Brazil	Amazonas	Homo sapiens	2024	PQ064763	PQ064764	PQ064765
LACENAM_ILMD_0026ARV	Brazil	Amazonas	Homo sapiens	2024	PQ064766	PQ064767	PQ064768
LACENAM_ILMD_0026MFS	Brazil	Amazonas	Homo sapiens	2023	PQ064769	PQ064770	PQ064771
LACENAM_ILMD_0026RLV	Brazil	Amazonas	Homo sapiens	2024	PQ064772	PQ064773	PQ064774
LACENAM_ILMD_0028	Brazil	Amazonas	Homo sapiens	2024	PQ064775	PQ064776	PQ064777
LACENAM_ILMD_0028WGF	Brazil	Amazonas	Homo sapiens	2023	PQ064778	PQ064779	PQ064780
LACENAM_ILMD_0029	Brazil	Amazonas	Homo sapiens	2024	PQ064781	PQ064782	PQ064783
LACENAM_ILMD_0029EBM	Brazil	Amazonas	Homo sapiens	2023	PQ064784	PQ064785	PQ064786
LACENAM_ILMD_0030	Brazil	Amazonas	Homo sapiens	2024	PQ064787	PQ064788	PQ064789
LACENAM_ILMD_0030ACN	Brazil	Amazonas	Homo sapiens	2024	PQ064790	PQ064791	PQ064792
LACENAM_ILMD_0030OPF	Brazil	Amazonas	Homo sapiens	2023	PQ064793	PQ064794	PQ064795
LACENAM_ILMD_0032	Brazil	Amazonas	Homo sapiens	2024	PQ064796	PQ064797	PQ064798
LACENAM_ILMD_0033	Brazil	Amazonas	Homo sapiens	2024	PQ064799	PQ064800	PQ064801
LACENAM_ILMD_0034	Brazil	Amazonas	Homo sapiens	2024	PQ064802	PQ064803	PQ064804

LACENAM_ILMD_0036GLF	Brazil	Amazonas	Homo sapiens	2024	PQ064805	PQ064806	PQ064807
LACENAM_ILMD_0038VNS	Brazil	Amazonas	Homo sapiens	2024	PQ064808	PQ064809	PQ064810
LACENAM_ILMD_0039IJM	Brazil	Amazonas	Homo sapiens	2023	PQ064811	PQ064812	PQ064813
LACENAM_ILMD_0041	Brazil	Amazonas	Homo sapiens	2024	PQ064814	PQ064815	PQ064816
LACENAM_ILMD_0042ESC	Brazil	Amazonas	Homo sapiens	2024	PQ064817	PQ064818	PQ064819
LACENAM_ILMD_0042MRO	Brazil	Amazonas	Homo sapiens	2024	PQ064820	PQ064821	PQ064822
LACENAM_ILMD_0044	Brazil	Amazonas	Homo sapiens	2024	PQ064823	PQ064824	PQ064825
LACENAM_ILMD_0046	Brazil	Amazonas	Homo sapiens	2024	PQ064826	PQ064827	PQ064828
LACENAM_ILMD_0047	Brazil	Amazonas	Homo sapiens	2024	PQ064829	PQ064830	PQ064831
LACENAM_ILMD_0048	Brazil	Amazonas	Homo sapiens	2024	PQ064832	PQ064833	PQ064834
LACENAM_ILMD_0048CJL	Brazil	Amazonas	Homo sapiens	2024	PQ064835	PQ064836	PQ064837
LACENAM_ILMD_0048DSM	Brazil	Amazonas	Homo sapiens	2024	PQ064838	PQ064839	PQ064840
LACENAM_ILMD_0054	Brazil	Amazonas	Homo sapiens	2024	PQ064841	PQ064842	PQ064843
LACENAM_ILMD_0054MDR	Brazil	Amazonas	Homo sapiens	2024	PQ064844	PQ064845	PQ064846
LACENAM_ILMD_0055LMS	Brazil	Amazonas	Homo sapiens	2024	PQ064847	PQ064848	PQ064849
LACENAM_ILMD_0056	Brazil	Amazonas	Homo sapiens	2024	PQ064850	PQ064851	PQ064852
LACENAM_ILMD_0081MSA	Brazil	Amazonas	Homo sapiens	2024	PQ064853	PQ064854	PQ064855
LACENAM_ILMD_0086	Brazil	Amazonas	Homo sapiens	2023	PQ064856	PQ064857	PQ064858
LACENAM_ILMD_0087	Brazil	Amazonas	Homo sapiens	2023	PQ064859	PQ064860	PQ064861
LACENAM_ILMD_0094	Brazil	Amazonas	Homo sapiens	2024	PQ064862	PQ064863	PQ064864
LACENAM_ILMD_0098ECA	Brazil	Amazonas	Homo sapiens	2024	PQ064865	PQ064866	PQ064867
LACENAM_ILMD_0098JS	Brazil	Amazonas	Homo sapiens	2023	PQ064868	PQ064869	PQ064870
LACENAM_ILMD_0099	Brazil	Amazonas	Homo sapiens	2023	PQ064871	PQ064872	PQ064873
LACENAM_ILMD_0102ABO	Brazil	Amazonas	Homo sapiens	2024	PQ064874	PQ064875	PQ064876
LACENAM_ILMD_0102VSV	Brazil	Amazonas	Homo sapiens	2023	PQ064877	PQ064878	PQ064879
LACENAM_ILMD_0103	Brazil	Amazonas	Homo sapiens	2023	PQ064880	PQ064881	PQ064882
LACENAM_ILMD_0103FHC	Brazil	Amazonas	Homo sapiens	2024	PQ064883	PQ064884	PQ064885
LACENAM_ILMD_0104	Brazil	Amazonas	Homo sapiens	2023	PQ064886	PQ064887	PQ064888
LACENAM_ILMD_0105	Brazil	Amazonas	Homo sapiens	2024	PQ064889	PQ064890	PQ064891
LACENAM_ILMD_0106	Brazil	Amazonas	Homo sapiens	2023	PQ064892	PQ064893	PQ064894
LACENAM_ILMD_0106ESC	Brazil	Amazonas	Homo sapiens	2024	PQ064895	PQ064896	PQ064897
LACENAM_ILMD_0107GDL	Brazil	Amazonas	Homo sapiens	2023	PQ064898	PQ064899	PQ064900
LACENAM_ILMD_0107IRM	Brazil	Amazonas	Homo sapiens	2024	PQ064901	PQ064902	PQ064903
LACENAM_ILMD_0107JMR	Brazil	Amazonas	Homo sapiens	2024	PQ064904	PQ064905	PQ064906
LACENAM_ILMD_0109	Brazil	Amazonas	Homo sapiens	2023	PQ064907	PQ064908	PQ064909
LACENAM_ILMD_0111	Brazil	Amazonas	Homo sapiens	2023	PQ064910	PQ064911	PQ064912
LACENAM_ILMD_0112	Brazil	Amazonas	Homo sapiens	2023	PQ064913	PQ064914	PQ064915
LACENAM_ILMD_0114JLC	Brazil	Amazonas	Homo sapiens	2023	PQ064916	PQ064917	PQ064918
LACENAM_ILMD_0114RLS	Brazil	Amazonas	Homo sapiens	2024	PQ064919	PQ064920	PQ064921
LACENAM_ILMD_0116	Brazil	Amazonas	Homo sapiens	2024	PQ064922	PQ064923	PQ064924
LACENAM_ILMD_0117	Brazil	Amazonas	Homo sapiens	2024	PQ064925	PQ064926	PQ064927

LACENAM_ILMD_0118	Brazil	Amazonas	Homo sapiens	2023	PQ064928	PQ064929	PQ064930
LACENAM_ILMD_0119	Brazil	Amazonas	Homo sapiens	2024	PQ064931	PQ064932	PQ064933
LACENAM_ILMD_0120	Brazil	Amazonas	Homo sapiens	2024	PQ064934	PQ064935	PQ064936
LACENAM_ILMD_0125	Brazil	Amazonas	Homo sapiens	2024	PQ064937	PQ064938	PQ064939
LACENAM_ILMD_0128	Brazil	Amazonas	Homo sapiens	2024	PQ064940	PQ064941	PQ064942
LACENAM_ILMD_0130	Brazil	Amazonas	Homo sapiens	2024	PQ064943	PQ064944	PQ064945
LACENAM_ILMD_0135MLC	Brazil	Amazonas	Homo sapiens	2024	PQ064946	PQ064947	PQ064948
LACENAM_ILMD_0138KCA	Brazil	Amazonas	Homo sapiens	2023	PQ064949	PQ064950	PQ064951
LACENAM_ILMD_0138MOP	Brazil	Amazonas	Homo sapiens	2024	PQ064952	PQ064953	PQ064954
LACENAM_ILMD_0138RMM	Brazil	Amazonas	Homo sapiens	2024	PQ064955	PQ064956	PQ064957
LACENAM_ILMD_0139	Brazil	Amazonas	Homo sapiens	2024	PQ064958	PQ064959	PQ064960
LACENAM_ILMD_0142	Brazil	Amazonas	Homo sapiens	2024	PQ064961	PQ064962	PQ064963
LACENAM_ILMD_0143	Brazil	Amazonas	Homo sapiens	2024	PQ064964	PQ064965	PQ064966
LACENAM_ILMD_0144	Brazil	Amazonas	Homo sapiens	2024	PQ064967	PQ064968	PQ064969
LACENAM_ILMD_0151	Brazil	Amazonas	Homo sapiens	2024	PQ064970	PQ064971	PQ064972
LACENAM_ILMD_0152MCB	Brazil	Amazonas	Homo sapiens	2024	PQ064973	PQ064974	PQ064975
LACENAM_ILMD_0153	Brazil	Amazonas	Homo sapiens	2024	PQ064976	PQ064977	PQ064978
LACENAM_ILMD_0154	Brazil	Amazonas	Homo sapiens	2024	PQ064979	PQ064980	PQ064981
LACENAM_ILMD_0155	Brazil	Amazonas	Homo sapiens	2024	PQ064982	PQ064983	PQ064984
LACENAM_ILMD_0157KSP	Brazil	Amazonas	Homo sapiens	2024	PQ064985	PQ064986	PQ064987
LACENAM_ILMD_0160	Brazil	Amazonas	Homo sapiens	2024	PQ064988	PQ064989	PQ064990
LACENAM_ILMD_0160WBS	Brazil	Amazonas	Homo sapiens	2024	PQ064991	PQ064992	PQ064993
LACENAM_ILMD_0165BSC	Brazil	Amazonas	Homo sapiens	2024	PQ064994	PQ064995	PQ064996
LACENAM_ILMD_0170VPM	Brazil	Amazonas	Homo sapiens	2024	PQ064997	PQ064998	PQ064999
LACENAM_ILMD_0175MFA	Brazil	Amazonas	Homo sapiens	2024	PQ065000	PQ065001	PQ065002
LACENAM_ILMD_0186TAM	Brazil	Amazonas	Homo sapiens	2024	PQ065003	PQ065004	PQ065005
LACENAM_ILMD_0187RCC	Brazil	Amazonas	Homo sapiens	2024	PQ065006	PQ065007	PQ065008
LACENAM_ILMD_0188MRL	Brazil	Amazonas	Homo sapiens	2024	PQ065009	PQ065010	PQ065011
LACENAM_ILMD_0190RAR	Brazil	Amazonas	Homo sapiens	2024	PQ065012	PQ065013	PQ065014
LACENAM_ILMD_0192ELL	Brazil	Amazonas	Homo sapiens	2024	PQ065015	PQ065016	PQ065017
LACENAM_ILMD_0248FSM	Brazil	Amazonas	Homo sapiens	2024	PQ065018	PQ065019	PQ065020
LACENAM_ILMD_0251AIM	Brazil	Amazonas	Homo sapiens	2024	PQ065021	PQ065022	PQ065023
LACENAM_ILMD_0258MSS	Brazil	Amazonas	Homo sapiens	2024	PQ065024	PQ065025	PQ065026
LACENAM_ILMD_0260MLS	Brazil	Amazonas	Homo sapiens	2024	PQ065027	PQ065028	PQ065029
LACENAM_ILMD_0262LGV	Brazil	Amazonas	Homo sapiens	2024	PQ065030	PQ065031	PQ065032
LACENAM_ILMD_0263SRA	Brazil	Amazonas	Homo sapiens	2024	PQ065033	PQ065034	PQ065035
LACENAM_ILMD_0265AMS	Brazil	Amazonas	Homo sapiens	2024	PQ065036	PQ065037	PQ065038
LACENAM_ILMD_0298ZMC	Brazil	Amazonas	Homo sapiens	2024	PQ065039	PQ065040	PQ065041
LACENAM_ILMD_0299MCS	Brazil	Amazonas	Homo sapiens	2024	PQ065042	PQ065043	PQ065044
LACENAM_ILMD_0301HSF	Brazil	Amazonas	Homo sapiens	2024	PQ065045	PQ065046	PQ065047
LACENAM_ILMD_0383ESF	Brazil	Amazonas	Homo sapiens	2024	PQ065048	PQ065049	PQ065050

LACENAM_ILMD_0411DSB	Brazil	Amazonas	Homo sapiens	2024	PQ065051	PQ065052	PQ065053
LACENAM_ILMD_0413ACB	Brazil	Amazonas	Homo sapiens	2024	PQ065054	PQ065055	PQ065056
LACENAM_ILMD_0417JOO	Brazil	Amazonas	Homo sapiens	2024	PQ065057	PQ065058	PQ065059
LACENAM_ILMD_0430MCM	Brazil	Amazonas	Homo sapiens	2024	PQ065060	PQ065061	PQ065062
LACENAM_ILMD_0433CSC	Brazil	Amazonas	Homo sapiens	2024	PQ065063	PQ065064	PQ065065
LACENAM_ILMD_0444ESC	Brazil	Amazonas	Homo sapiens	2024	PQ065066	PQ065067	PQ065068
LACENAM_ILMD_0446AOM	Brazil	Amazonas	Homo sapiens	2024	PQ065069	PQ065070	PQ065071
LACENAM_ILMD_0449ASV	Brazil	Amazonas	Homo sapiens	2024	PQ065072	PQ065073	PQ065074
LACENAM_ILMD_0452OSS	Brazil	Amazonas	Homo sapiens	2024	PQ065075	PQ065076	PQ065077
LACENAM_ILMD_0454ASB	Brazil	Amazonas	Homo sapiens	2024	PQ065078	PQ065079	PQ065080
LACENAM_ILMD_0454ESS	Brazil	Amazonas	Homo sapiens	2024	PQ065081	PQ065082	PQ065083
LACENAM_ILMD_0461SLS	Brazil	Amazonas	Homo sapiens	2024	PQ065084	PQ065085	PQ065086
LACENAM_ILMD_0462CCL	Brazil	Amazonas	Homo sapiens	2024	PQ065087	PQ065088	PQ065089
LACENAM_ILMD_0487LFB	Brazil	Amazonas	Homo sapiens	2024	PQ065090	PQ065091	PQ065092
LACENAM_ILMD_0488ENB	Brazil	Amazonas	Homo sapiens	2024	PQ065093	PQ065094	PQ065095
LACENAM_ILMD_0489SGT	Brazil	Amazonas	Homo sapiens	2024	PQ065096	PQ065097	PQ065098
LACENAM_ILMD_0504DBC	Brazil	Amazonas	Homo sapiens	2024	PQ065099	PQ065100	PQ065101
LACENAM_ILMD_0505LMM	Brazil	Amazonas	Homo sapiens	2024	PQ065102	PQ065103	PQ065104
LACENAM_ILMD_0508JSC	Brazil	Amazonas	Homo sapiens	2024	PQ065105	PQ065106	PQ065107
LACENAM_ILMD_0511GCS	Brazil	Amazonas	Homo sapiens	2024	PQ065108	PQ065109	PQ065110
LACENAM_ILMD_0512GRB	Brazil	Amazonas	Homo sapiens	2024	PQ065111	PQ065112	PQ065113
LACENAM_ILMD_0514DMG	Brazil	Amazonas	Homo sapiens	2024	PQ065114	PQ065115	PQ065116
LACENAM_ILMD_0515KTB	Brazil	Amazonas	Homo sapiens	2024	PQ065117	PQ065118	PQ065119
LACENAM_ILMD_0518ACM	Brazil	Amazonas	Homo sapiens	2024	PQ065120	PQ065121	PQ065122
LACENAM_ILMD_0544ASM	Brazil	Amazonas	Homo sapiens	2024	PQ065123	PQ065124	PQ065125
LACENAM_ILMD_0546LBO	Brazil	Amazonas	Homo sapiens	2024	PQ065126	PQ065127	PQ065128
LACENAM_ILMD_0548CFM	Brazil	Amazonas	Homo sapiens	2024	PQ065129	PQ065130	PQ065131
LACENAM_ILMD_0553APS	Brazil	Amazonas	Homo sapiens	2024	PQ065132	PQ065133	PQ065134
LACENAM_ILMD_0572MNA	Brazil	Amazonas	Homo sapiens	2024	PQ065135	PQ065136	PQ065137
LACENAM_ILMD_0600MJO	Brazil	Amazonas	Homo sapiens	2024	PQ065138	PQ065139	PQ065140
LACENAM_ILMD_0602GVL	Brazil	Amazonas	Homo sapiens	2024	PQ065141	PQ065142	PQ065143
LACENAM_ILMD_0646RRM	Brazil	Amazonas	Homo sapiens	2024	PQ065144	PQ065145	PQ065146
LACENAM_ILMD_0671AB	Brazil	Amazonas	Homo sapiens	2024	PQ065147	PQ065148	PQ065149
LACENAM_ILMD_0737JSC	Brazil	Amazonas	Homo sapiens	2023	PQ065150	PQ065151	PQ065152
LACENAM_ILMD_0752DSF	Brazil	Amazonas	Homo sapiens	2024	PQ065153	PQ065154	PQ065155
LACENAM_ILMD_0777DSS	Brazil	Amazonas	Homo sapiens	2023	PQ065156	PQ065157	PQ065158
LACENAM_ILMD_0779MCA	Brazil	Amazonas	Homo sapiens	2024	PQ065159	PQ065160	PQ065161
LACENAM_ILMD_0786FLG	Brazil	Amazonas	Homo sapiens	2024	PQ065162	PQ065163	PQ065164
LACENAM_ILMD_0788RSC	Brazil	Amazonas	Homo sapiens	2024	PQ065165	PQ065166	PQ065167
LACENAM_ILMD_0832JSM	Brazil	Amazonas	Homo sapiens	2024	PQ065168	PQ065169	PQ065170
LACENAM_ILMD_1109RBL	Brazil	Amazonas	Homo sapiens	2024	PQ065171	PQ065172	PQ065173

LACENAM_ILMD_1110JHT	Brazil	Amazonas	Homo sapiens	2024	PQ065174	PQ065175	PQ065176
LACENAM_ILMD_1117EBL	Brazil	Amazonas	Homo sapiens	2024	PQ065177	PQ065178	PQ065179
LACENAM_ILMD_1118RET	Brazil	Amazonas	Homo sapiens	2024	PQ065180	PQ065181	PQ065182
LACENAM_ILMD_1173PRF	Brazil	Amazonas	Homo sapiens	2024	PQ065183	PQ065184	PQ065185
LACENAM_ILMD_1176AFB	Brazil	Amazonas	Homo sapiens	2024	PQ065186	PQ065187	PQ065188
LACENAM_ILMD_1189LMA	Brazil	Amazonas	Homo sapiens	2024	PQ065189	PQ065190	PQ065191
LACENAM_ILMD_1197FSM	Brazil	Amazonas	Homo sapiens	2024	PQ065192	PQ065193	PQ065194
LACENAM_ILMD_1200MNA	Brazil	Amazonas	Homo sapiens	2024	PQ065195	PQ065196	PQ065197
LACENAM_ILMD_1203FAR	Brazil	Amazonas	Homo sapiens	2024	PQ065198	PQ065199	PQ065200
LACENAM_ILMD_1208CMC	Brazil	Amazonas	Homo sapiens	2024	PQ065201	PQ065202	PQ065203
LACENAM_ILMD_1210RSR	Brazil	Amazonas	Homo sapiens	2024	PQ065204	PQ065205	PQ065206
LACENAM_ILMD_1212LRS	Brazil	Amazonas	Homo sapiens	2024	PQ065207	PQ065208	PQ065209
LACENAM_ILMD_1233WMC	Brazil	Amazonas	Homo sapiens	2024	PQ065210	PQ065211	PQ065212
LACENAM_ILMD_1234MFL	Brazil	Amazonas	Homo sapiens	2024	PQ065213	PQ065214	PQ065215
LACENAM_ILMD_1268JSS	Brazil	Amazonas	Homo sapiens	2024	PQ065216	PQ065217	PQ065218
LACENAM_ILMD_1532MSA	Brazil	Amazonas	Homo sapiens	2023	PQ065219	PQ065220	PQ065221
LACENAM_ILMD_2767HSR	Brazil	Amazonas	Homo sapiens	2023	PQ065222	PQ065223	PQ065224
LACENAM_ILMD_2783WSA	Brazil	Amazonas	Homo sapiens	2023	PQ065225	PQ065226	PQ065227
LACENAM_ILMD_2826NLL	Brazil	Amazonas	Homo sapiens	2023	PQ065228	PQ065229	PQ065230
LACENAM_ILMD_3034LOG	Brazil	Amazonas	Homo sapiens	2023	PQ065231	PQ065232	PQ065233
LACENAM_ILMD_3036CMS	Brazil	Amazonas	Homo sapiens	2023	PQ065234	PQ065235	PQ065236
LACENAM_ILMD_3039JLS	Brazil	Amazonas	Homo sapiens	2023	PQ065237	PQ065238	PQ065239
LACENAM_ILMD_3040DCS	Brazil	Amazonas	Homo sapiens	2023	PQ065240	PQ065241	PQ065242
LACENAM_ILMD_3048MAS	Brazil	Amazonas	Homo sapiens	2023	PQ065243	PQ065244	PQ065245
LACENAM_ILMD_3070EPP	Brazil	Amazonas	Homo sapiens	2023	PQ065246	PQ065247	PQ065248
LACENAM_ILMD_3078EBS	Brazil	Amazonas	Homo sapiens	2023	PQ065249	PQ065250	PQ065251
LACENAM_ILMD_3079VHF	Brazil	Amazonas	Homo sapiens	2023	PQ065252	PQ065253	PQ065254
LACENAM_ILMD_3090ASS	Brazil	Amazonas	Homo sapiens	2023	PQ065255	PQ065256	PQ065257
LACENAM_ILMD_3091LGS	Brazil	Amazonas	Homo sapiens	2023	PQ065258	PQ065259	PQ065260
LACENAM_ILMD_3093APL	Brazil	Amazonas	Homo sapiens	2023	PQ065261	PQ065262	PQ065263
LACENAM_ILMD_3097VFR	Brazil	Amazonas	Homo sapiens	2023	PQ065264	PQ065265	PQ065266
LACENAM_ILMD_3099LSR	Brazil	Amazonas	Homo sapiens	2023	PQ065267	PQ065268	PQ065269
LACENAM_ILMD_3120APA	Brazil	Amazonas	Homo sapiens	2023	PQ065270	PQ065271	PQ065272
LACENAM_ILMD_3121DRS	Brazil	Amazonas	Homo sapiens	2023	PQ065273	PQ065274	PQ065275
LACENAM_ILMD_3123RAS	Brazil	Amazonas	Homo sapiens	2023	PQ065276	PQ065277	PQ065278
LACENAM_ILMD_3126ETO	Brazil	Amazonas	Homo sapiens	2023	PQ065279	PQ065280	PQ065281
LACENAM_ILMD_3127DFS	Brazil	Amazonas	Homo sapiens	2023	PQ065282	PQ065283	PQ065284
LACENAM_ILMD_3129JWA	Brazil	Amazonas	Homo sapiens	2023	PQ065285	PQ065286	PQ065287
LACENAM_ILMD_3134JVC	Brazil	Amazonas	Homo sapiens	2023	PQ065288	PQ065289	PQ065290
LACENAM_ILMD_3145LMV	Brazil	Amazonas	Homo sapiens	2023	PQ065291	PQ065292	PQ065293
LACENAM_ILMD_3210EES	Brazil	Amazonas	Homo sapiens	2023	PQ065294	PQ065295	PQ065296

LACENAM_ILMD_3212EDS	Brazil	Amazonas	Homo sapiens	2023	PQ065297	PQ065298	PQ065299
LACENAM_ILMD_3216VSV	Brazil	Amazonas	Homo sapiens	2023	PQ065300	PQ065301	PQ065302
LACENAM_ILMD_3218MPG	Brazil	Amazonas	Homo sapiens	2023	PQ065303	PQ065304	PQ065305
LACENAM_ILMD_3219MTM	Brazil	Amazonas	Homo sapiens	2023	PQ065306	PQ065307	PQ065308
LACENAM_ILMD_3228ZCF	Brazil	Amazonas	Homo sapiens	2023	PQ065309	PQ065310	PQ065311
LACENAM_ILMD_3229FMC	Brazil	Amazonas	Homo sapiens	2023	PQ065312	PQ065313	PQ065314
LACENAM_ILMD_3230FPP	Brazil	Amazonas	Homo sapiens	2023	PQ065315	PQ065316	PQ065317
LACENAM_ILMD_3234ERL	Brazil	Amazonas	Homo sapiens	2023	PQ065318	PQ065319	PQ065320
LACENAM_ILMD_3236GAB	Brazil	Amazonas	Homo sapiens	2023	PQ065321	PQ065322	PQ065323
LACENAM_ILMD_3237FES	Brazil	Amazonas	Homo sapiens	2023	PQ065324	PQ065325	PQ065326
LACENAM_ILMD_3238CGR	Brazil	Amazonas	Homo sapiens	2023	PQ065327	PQ065328	PQ065329
LACENAM_ILMD_3239VNR	Brazil	Amazonas	Homo sapiens	2023	PQ065330	PQ065331	PQ065332
LACENAM_ILMD_3240RFA	Brazil	Amazonas	Homo sapiens	2023	PQ065333	PQ065334	PQ065335
LACENAM_ILMD_3245ESL	Brazil	Amazonas	Homo sapiens	2023	PQ065336	PQ065337	PQ065338
LACENAM_ILMD_3246KMP	Brazil	Amazonas	Homo sapiens	2023	PQ065339	PQ065340	PQ065341
LACENAM_ILMD_3247ARA	Brazil	Amazonas	Homo sapiens	2023	PQ065342	PQ065343	PQ065344
LACENAM_ILMD_3251RAS	Brazil	Amazonas	Homo sapiens	2023	PQ065345	PQ065346	PQ065347
LACENAM_ILMD_3252EFS	Brazil	Amazonas	Homo sapiens	2023	PQ065348	PQ065349	PQ065350
LACENAM_ILMD_3255RZS	Brazil	Amazonas	Homo sapiens	2023	PQ065351	PQ065352	PQ065353
LACENAM_ILMD_3265AMC	Brazil	Amazonas	Homo sapiens	2023	PQ065354	PQ065355	PQ065356
LACENAM_ILMD_3268JRS	Brazil	Amazonas	Homo sapiens	2023	PQ065357	PQ065358	PQ065359
LACENAM_ILMD_3269RAN	Brazil	Amazonas	Homo sapiens	2023	PQ065360	PQ065361	PQ065362
LACENAM_ILMD_3275FSP	Brazil	Amazonas	Homo sapiens	2023	PQ065363	PQ065364	PQ065365
LACENAM_ILMD_3279HAN	Brazil	Amazonas	Homo sapiens	2023	PQ065366	PQ065367	PQ065368
LACENAM_ILMD_3280CHC	Brazil	Amazonas	Homo sapiens	2023	PQ065369	PQ065370	PQ065371
LACENAM_ILMD_3285ALS	Brazil	Amazonas	Homo sapiens	2023	PQ065372	PQ065373	PQ065374
LACENAM_ILMD_3286ESB	Brazil	Amazonas	Homo sapiens	2023	PQ065375	PQ065376	PQ065377
LACENAM_ILMD_3292WOF	Brazil	Amazonas	Homo sapiens	2023	PQ065378	PQ065379	PQ065380
LACENAM_ILMD_3293MGG	Brazil	Amazonas	Homo sapiens	2023	PQ065381	PQ065382	PQ065383
LACENAM_ILMD_3303CSL	Brazil	Amazonas	Homo sapiens	2023	PQ065384	PQ065385	PQ065386
LACENAM_ILMD_3307MBS	Brazil	Amazonas	Homo sapiens	2023	PQ065387	PQ065388	PQ065389
LACENAM_ILMD_3319FHS	Brazil	Amazonas	Homo sapiens	2023	PQ065390	PQ065391	PQ065392
LACENAM_ILMD_3322RFS	Brazil	Amazonas	Homo sapiens	2023	PQ065393	PQ065394	PQ065395
LACENAM_ILMD_3323ACA	Brazil	Amazonas	Homo sapiens	2023	PQ065396	PQ065397	PQ065398
LACENAM_ILMD_3330GGF	Brazil	Amazonas	Homo sapiens	2023	PQ065399	PQ065400	PQ065401
LACENAM_ILMD_3331NM	Brazil	Amazonas	Homo sapiens	2023	PQ065402	PQ065403	PQ065404
LACENAM_ILMD_3333VMA	Brazil	Amazonas	Homo sapiens	2023	PQ065405	PQ065406	PQ065407
LACENAM_ILMD_3334ASF	Brazil	Amazonas	Homo sapiens	2023	PQ065408	PQ065409	PQ065410
LACENAM_ILMD_3335ANL	Brazil	Amazonas	Homo sapiens	2023	PQ065411	PQ065412	PQ065413
LACENAM_ILMD_3337ISP	Brazil	Amazonas	Homo sapiens	2023	PQ065414	PQ065415	PQ065416
LACENAM_ILMD_3339DOA	Brazil	Amazonas	Homo sapiens	2023	PQ065417	PQ065418	PQ065419

LACENAM_ILMD_3345HTO	Brazil	Amazonas	Homo sapiens	2023	PQ065420	PQ065421	PQ065422
LACENAM_ILMD_3351JSV	Brazil	Amazonas	Homo sapiens	2023	PQ065423	PQ065424	PQ065425
LACENAM_ILMD_3352LBB	Brazil	Amazonas	Homo sapiens	2023	PQ065426	PQ065427	PQ065428
LACENAM_ILMD_3354MEA	Brazil	Amazonas	Homo sapiens	2023	PQ065429	PQ065430	PQ065431
LACENAM_ILMD_3359ROS	Brazil	Amazonas	Homo sapiens	2023	PQ065432	PQ065433	PQ065434
LACENAM_ILMD_3369ESS	Brazil	Amazonas	Homo sapiens	2023	PQ065435	PQ065436	PQ065437
LACENAM_ILMD_3372ALM	Brazil	Amazonas	Homo sapiens	2023	PQ065438	PQ065439	PQ065440
LACENAM_ILMD_3375ECR	Brazil	Amazonas	Homo sapiens	2023	PQ065441	PQ065442	PQ065443
LACENAM_ILMD_3376EST	Brazil	Amazonas	Homo sapiens	2023	PQ065444	PQ065445	PQ065446
LACENAM_ILMD_3377DSL	Brazil	Amazonas	Homo sapiens	2023	PQ065447	PQ065448	PQ065449
LACENAM_ILMD_3381RRM	Brazil	Amazonas	Homo sapiens	2023	PQ065450	PQ065451	PQ065452
LACENAM_ILMD_3383ALS	Brazil	Amazonas	Homo sapiens	2023	PQ065453	PQ065454	PQ065455
LACENAM_ILMD_3388ROB	Brazil	Amazonas	Homo sapiens	2023	PQ065456	PQ065457	PQ065458
LACENAM_ILMD_3400ABN	Brazil	Amazonas	Homo sapiens	2023	PQ065459	PQ065460	PQ065461
LACENAM_ILMD_6029TMV	Brazil	Amazonas	Homo sapiens	2023	PQ065462	PQ065463	PQ065464
LACENAM_ILMD_9338AIVP	Brazil	Amazonas	Homo sapiens	2023	PQ065465	PQ065466	PQ065467
LACENAM_ILMD_9476SCS	Brazil	Amazonas	Homo sapiens	2023	PQ065468	PQ065469	PQ065470
LACENAM_ILMD_9532DRS	Brazil	Amazonas	Homo sapiens	2023	PQ065471	PQ065472	PQ065473
LACENAM_ILMD_9534KSO	Brazil	Amazonas	Homo sapiens	2023	PQ065474	PQ065475	PQ065476
LACENAM_ILMD_9535JCS	Brazil	Amazonas	Homo sapiens	2023	PQ065477	PQ065478	PQ065479
LACENPR_ILMD_9982IOM	Brazil	Acre	Homo sapiens	2023	PQ065480	PQ065481	PQ065482
LVM_ILMD_ARB-34	Brazil	Acre	Homo sapiens	2023	PQ065483	PQ065484	PQ065485
LVM_ILMD_ZDC-594	Brazil	Rondonia	Homo sapiens	2023	PQ065486	PQ065487	PQ065488
LVM_ILMD_ZDC-622	Brazil	Rondonia	Homo sapiens	2023	PQ065489	PQ065490	PQ065491
hOROV/Brazil/PE-IAM4637/2024	Brazil	Pernambuco	Homo sapiens	2024	PQ073181	PQ073182	PQ073183
hOROV/Brazil/PE-IAM4578/2024	Brazil	Pernambuco	Homo sapiens	2024	PQ073184	PQ073185	PQ073186
BeAn 423380	Brazil	-	Nasua nasua	1984	NC_043578	NC_043577	NC_043576

References

1. Proenca-Modena JL, Hyde JL, Sesti-Costa R, et al. Interferon-Regulatory Factor 5-Dependent Signaling Restricts Orthobunyavirus Dissemination to the Central Nervous System. *J Virol* 2016; **90**(1): 189-205.
2. de Souza Luna LK, Rodrigues AH, Santos RI, et al. Oropouche virus is detected in peripheral blood leukocytes from patients. *J Med Virol* 2017; **89**(6): 1108-11.
3. Naveca FG, Nascimento VAD, Souza VC, Nunes BTD, Rodrigues DSG, Vasconcelos PFDC. Multiplexed reverse transcription real-time polymerase chain reaction for simultaneous detection of Mayaro, Oropouche, and Oropouche-like viruses. *Mem Inst Oswaldo Cruz* 2017; **112**(7): 510-3.
4. Lanciotti RS, Kosoy OL, Laven JJ, et al. Chikungunya virus in US travelers returning from India, 2006. *Emerg Infect Dis* 2007; **13**(5): 764-7.
5. Johnson BW, Russell BJ, Lanciotti RS. Serotype-specific detection of dengue viruses in a fourplex real-time reverse transcriptase PCR assay. *J Clin Microbiol* 2005; **43**(10): 4977-83.

6. Callahan JD, Wu SJ, Dion-Schultz A, et al. Development and evaluation of serotype- and group-specific fluorogenic reverse transcriptase PCR (TaqMan) assays for dengue virus. *J Clin Microbiol* 2001; **39**(11): 4119-24.
7. Waggoner JJ, Rojas A, Mohamed-Hadley A, de Guillén YA, Pinsky BA. Real-time RT-PCR for Mayaro virus detection in plasma and urine. *J Clin Virol* 2018; **98**: 1-4.
8. Proenca-Modena JL, Sesti-Costa R, Pinto AK, et al. Oropouche virus infection and pathogenesis are restricted by MAVS, IRF-3, IRF-7, and type I interferon signaling pathways in nonmyeloid cells. *J Virol* 2015; **89**(9): 4720-37.
9. Claro IM, Ramundo MS, Coletti TM, et al. Rapid viral metagenomics using SMART-9N amplification and nanopore sequencing. *Wellcome Open Res* 2021; **6**: 241.
10. Li H. Minimap2: pairwise alignment for nucleotide sequences. *Bioinformatics* 2018; **34**(18): 3094-100.
11. Li H, Handsaker B, Wysoker A, et al. The Sequence Alignment/Map format and SAMtools. *Bioinformatics* 2009; **25**(16): 2078-9.
12. Danecek P, Bonfield JK, Liddle J, et al. Twelve years of SAMtools and BCFtools. *Gigascience* 2021; **10**(2).
13. De Coster W, D'Hert S, Schultz DT, Cruts M, Van Broeckhoven C. NanoPack: visualizing and processing long-read sequencing data. *Bioinformatics* 2018; **34**(15): 2666-9.
14. Katoh K, Standley DM. MAFFT multiple sequence alignment software version 7: improvements in performance and usability. *Mol Biol Evol* 2013; **30**(4): 772-80.
15. Nguyen LT, Schmidt HA, von Haeseler A, Minh BQ. IQ-TREE: a fast and effective stochastic algorithm for estimating maximum-likelihood phylogenies. *Mol Biol Evol* 2015; **32**(1): 268-74.
16. Kalyaanamoorthy S, Minh BQ, Wong TKF, von Haeseler A, Jermiin LS. ModelFinder: fast model selection for accurate phylogenetic estimates. *Nat Methods* 2017; **14**(6): 587-9.
17. Tamura K, Nei M, Kumar S. Prospects for inferring very large phylogenies by using the neighbor-joining method. *Proc Natl Acad Sci U S A* 2004; **101**(30): 11030-5.
18. Martin DP, Varsani A, Roumagnac P, et al. RDP5: a computer program for analyzing recombination in, and removing signals of recombination from, nucleotide sequence datasets. *Virus Evol* 2021; **7**(1): veaa087.
19. Kumar S, Stecher G, Tamura K. MEGA7: Molecular Evolutionary Genetics Analysis Version 7.0 for Bigger Datasets. *Mol Biol Evol* 2016; **33**(7): 1870-4.
